# Asymmetric effects of activating and inactivating cortical interneurons

**DOI:** 10.7554/eLife.18383

**Published:** 2016-10-10

**Authors:** Elizabeth AK Phillips, Andrea R Hasenstaub

**Affiliations:** 1 Neuroscience Graduate Program, University of California, San Francisco, San Francisco, United States; 2 Center for Integrative Neuroscience, University of California San Francisco, San Francisco, United States; 3 Coleman Memorial Laboratory, University of California, San Francisco, San Francisco, United States; 4 Department of Otolaryngology-Head and Neck Surgery, University of California, San Francisco, San Francisco, United States; Harvard University, United States

**Keywords:** optogenetics, interneurons, cortex, Mouse

## Abstract

Bidirectional manipulations – activation and inactivation – are widely used to identify the functions supported by specific cortical interneuron types. Implicit in much of this work is the notion that tonic activation and inactivation will both produce valid, internally consistent insights into interneurons’ computational roles. Here, using single-unit recordings in auditory cortex of awake mice, we show that this may not generally hold true. Optogenetically manipulating somatostatin-positive (Sst+) or parvalbumin-positive (Pvalb+) interneurons while recording tone-responses showed that Sst+ inactivation increased response gain, while Pvalb+ inactivation weakened tuning and decreased information transfer, implying that these neurons support delineable computational functions. But activating Sst+ and Pvalb+ interneurons revealed no such differences. We used a simple network model to understand this asymmetry, and showed how relatively small changes in key parameters, such as spontaneous activity or strength of the light manipulation, determined whether activation and inactivation would produce consistent or paradoxical conclusions regarding interneurons’ computational functions.

**DOI:** http://dx.doi.org/10.7554/eLife.18383.001

## eLife digest

The brain processes information through the activity of many different types of neurons. A common assumption is that we can work out the role of each type of neuron by increasing or decreasing its activity experimentally and observing the consequences. Imagine, for example, that increasing the activity of a group of neurons boosts the formation of memories, whereas decreasing their activity has the opposite effect. Can we conclude that the natural role of those neurons is to support memory formation?

This question has become increasingly pertinent in recent years, as advances in molecular genetic techniques such as optogenetics have made it easier to experimentally manipulate the activity of neurons. However, the ease with which these experiments can be done masks how difficult it can be to interpret the results.

To test the assumption that dialing a neuron’s activity up or down provides a straightforward readout of its role in the brain, Phillips and Hasenstaub used optogenetics to inactivate two types of neurons in an area of the mouse brain responsible for processing sound. Inactivating each cell type produced different outcomes. The standard way of interpreting these results is to conclude that the neurons must therefore have different, separable roles. However, using optogenetics to increase the activity of the neurons revealed no such differences in the neurons’ roles.

Using a computer model, Phillips and Hasenstaub showed that relatively small changes in factors such as the spontaneous activity of the neurons can affect the outcome of the experiments. Under certain circumstances, the two types of neurons respond in the same way to activation and inactivation; under other circumstances, they respond differently.

The results presented by Phillips and Hasenstaub remind us that the conclusions we draw about a neuron’s role can be sensitive to how the cell was manipulated. Future studies should address how other aspects of experimental manipulations, such as their timing or pattern, affect the apparent behavior of neurons.

**DOI:** http://dx.doi.org/10.7554/eLife.18383.002

## Introduction

Throughout the neocortex, inhibition shapes the gain and tuning of sensory responses (Kyriazi et al., 1996; Tsumoto and Sato, 1985; Wang et al., 2000), controls the precise timing of evoked action potentials (Akiyama et al., 2004; Gabernet et al., 2005; Hasenstaub et al., 2005; Poo and Isaacson, 2009; Wehr and Zador, 2003), and can coordinate rhythmic activity, which gates sensory responses and modulates information processing (Cardin et al., 2009; Deans et al., 2001; Hasenstaub et al., 2005; Sohal et al., 2009). Although they comprise a minority of cells in the cortex, the GABAergic interneurons that provide this inhibition are strikingly diverse (rev. [Petilla Interneuron Nomenclature et al., 2008]): unique combinations of gene expression, electrophysiological properties, morphology, and connectivity define multiple interneuron types, and likely allow each interneuron type to exert a unique mode of control over cortical function.

A longstanding goal in sensory neuroscience has been to establish how, if at all, interneuron types differ in their contributions to normal cortical function. However, isolating each subtype to causally determine its contributions to cortical processing has been hindered by the limited repertoire of techniques available. Recent advances in molecular techniques, such as optogenetics (Boyden et al., 2005; Zhang et al., 2011), chemogenetics (Armbruster et al., 2007), and more recently magnetogenetics (Wheeler et al., 2016), have transcended traditional methods of probing cell function, enabling reversible and selective activation or inactivation of interneuron firing in vivo. These tools have been used extensively to identify the particular functions each interneuron subtype serves within the cortex (Adesnik et al., 2012; Aizenberg et al., 2015; Atallah et al., 2012; Cardin et al., 2009; Cottam et al., 2013; Lee et al., 2012; Natan et al., 2015; Seybold et al., 2015; Sohal et al., 2009; Wilson et al., 2012; Xu et al., 2013). An implicit assumption underlying much of this work is that one can directly read out a cell type’s function, and infer its specialized operations, by tonically modulating its firing down or up: in other words, that the response features or computations weakened by inactivating a cell population are specifically those that the cell population normally supports, that these features will be strengthened when the same population is activated, and that the natural differences in cortical function between cell populations may be read out, and interpreted, using these same strategies.

Can the abundance of recent causal data be interpreted in such a straightforward way? Here, in line with the broader goal of establishing the ways in which we can use causal strategies to gain insight into interneuron function, we tested this assumption by manipulating either of the two main families of cortical interneurons – somatostatin-positive (Sst+) or parvalbumin-positive (Pvalb+) interneurons – while recording neural responses to tones in the auditory cortex of awake mice. We found that inactivation of Sst+ interneurons increased response gain, while inactivation of Pvalb+ interneurons weakened tuning and decreased information transfer, implying that these two neuron types support specific, separable functions in the auditory cortex. Yet activating Sst+ and Pvalb+ interneurons revealed no such differences. We used a simple feedforward model to understand this asymmetry, and found that relatively small changes in key parameters, such as baseline activity, neural thresholds, or the strength of the light manipulation, determined whether activation and inactivation would produce internally consistent conclusions regarding interneurons’ computational functions. This implies that seemingly minor experimental details can qualitatively change the readout of a neural population’s role in computation, and that the conclusions we draw regarding neuronal function can be influenced, even distorted, by the precise way in which the neuronal populations are manipulated.

## Results

### Optogenetic inactivation of Sst+ or Pvalb+ interneurons

To produce mice in which we could inactivate Sst+ or Pvalb+ interneurons, we crossed mouse strains that express Cre-recombinase under control of the Sst or Pvalb promoter (Taniguchi et al., 2011) with the Ai35 strain, in which expression of Arch-GFP is Cre-dependent (Madisen et al., 2012) (referred to as Arch/Sst and Arch/Pvalb mice, respectively). In Arch/Sst mice, the percentage of GFP labeled neurons that showed Sst fluorescence (i.e., specificity) was 89 ± 4%, and the percentage of Sst labeled neurons that were GFP-labeled (i.e., efficiency) was 91 ± 2%, and in Arch/Pvalb mice the specificity was 93 ± 2% and the efficiency was 89 ± 7%, consistent with previous reports (Adesnik et al., 2012; Cardin et al., 2009; Kerlin et al., 2010; Kvitsiani et al., 2013; Pfeffer et al., 2013; Sohal et al., 2009; Taniguchi et al., 2011) (Figure 1—figure supplement 1).

**Figure 1.**
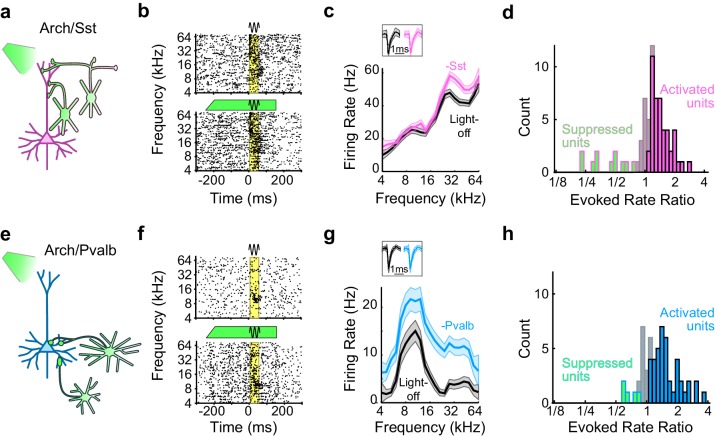
Optogenetic inactivation of Sst+ or Pvalb+ interneurons. (**a**) Schematic of optogenetic manipulation in Arch/Sst mice in which green light directly hyperpolarizes Sst+ cells (green cells). (**b**) Rasters of tone-evoked action potentials for a representative indirectly activated unit without (top) and with (bottom) inactivation of Sst+ cells. The black sine wave represents the duration of the sound, the green bar represents the duration and power of the light, and the yellow bar indicates the response period used to construct frequency tuning curves (FTCs). (**c**) FTCs (mean ± SEMs) derived from tone-evoked firing rates (FRs) without (black) and with (pink) inactivation of Sst+ cells for the representative unit in (**b**). Inset shows unit waveforms on trials without (black) and with (pink) inactivation of Sst+ cells. (**d**) Distribution of the light-on to light-off tone-evoked FR ratios during the response period for frequency-tuned units. Green bars with pink outline indicate units with significantly suppressed FRs (n = 11 of 70 units), grayish pink bars indicate units with no significant change in FR (n = 15 of 70 units), and pink bars with black outline indicate units with significantly increased FRs (n = 44 of 70 units). (**e–h**) As (**a–b**), but in Arch/Pvalb mice, in which green light directly hyperpolarizes Pvalb+ cells (**e**). (**f**,**g**) show the rasters and FTCs of a representative, indirectly-activated unit with and without inactivation of Pvalb+ cells. In (**h**), green bars with light blue outline indicate units with significantly suppressed FRs (n = 5 of 59 units), grayish blue bars indicate units with no significant change in FR (n = 13 of 59 units), and light blue bars with black outline indicate units with significantly increased FRs (n = 41 of 59 units). **DOI:** http://dx.doi.org/10.7554/eLife.18383.003

We placed these transgenic mice on a spherical treadmill and used a 16-channel linear probe to record from single units in auditory cortex while the animals were awake, head-fixed, and passively listening to pure-tone acoustic stimuli. On randomly interleaved trials the tone was paired with green (532 nm) light, delivered through a fiber optic placed just above the surface of the cortex, to reduce interneuron activity during the pre-stimulus and response periods (Figure 1a,b,e,f). For each recorded unit we generated iso-intensity frequency tuning curves (FTCs) in both the light-off and light-on conditions by measuring the firing rates (FRs) in the 50 ms period after tone-response onset as a function of frequency (Figure 1c,g). In these mice, illumination of the cortical surface with green light had a range of effects on sound-evoked FRs, spontaneous FRs, and the signal-to-noise ratio (SNR: defined 6as the ratio between sound-evoked FR and spontaneous FR) (Figure 1b,f; Figure 1—figure supplement 2). Of the units with frequency-tuned responses (see Materials and methods; Arch/Sst: n = 70 of 96 units; Arch/Pvalb: n = 59 of 76 units), the majority had sound-evoked FRs that were significantly increased by light (Arch/Sst: n = 44 of 70 units; Arch/Pvalb: n = 41 of 59 units); a minority had responses that were significantly suppressed by light (Arch/Sst: n = 11 of 70 units; Arch/Pvalb: n = 5 of 59 units); and the remainder had responses with no significant change (Arch/Sst: n = 15 of 70 units; Arch/Pvalb: n = 13 of 59 units) (Figure 1d,h). A small fraction of units with no significant change to their tone responses nonetheless showed significant changes to their baseline activity (Arch/Sst: n = 6 of 15; Arch/Pvalb: n = 4 of 13).

**Figure 1—figure supplement 1.**
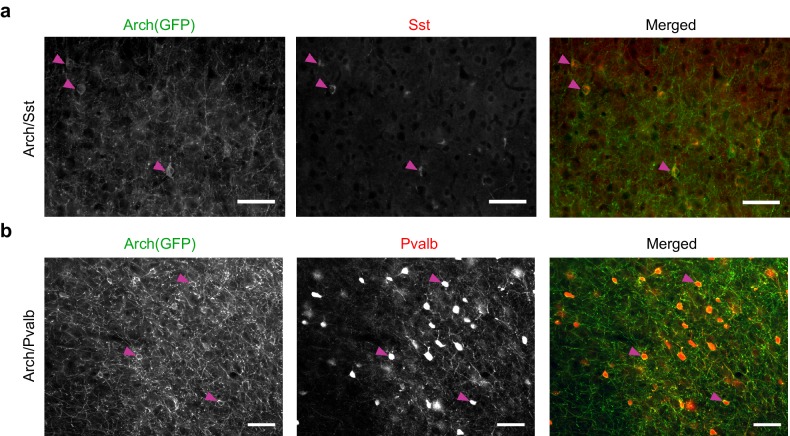
Arch(GFP) co-localizes with somatostatin in Arch/Sst mice and with parvalbumin in Arch/Pvalb mice. (**a**) Immunostaining of a brain slice from an Arch/Sst mouse. (Left) Grayscale image of GFP stain. (Middle) Grayscale image of somatostatin stain. (Right) Merged image of GFP (green) and somatostatin (red) stains. Magenta arrows indicate three examples of putative Sst+ cells. Note that red and green somata mostly overlap. Scale bars are 50 microns. (**b**) Immunostaining of a brain slice from an Arch/Pvalb mouse. (Left) Grayscale image of GFP stain. (Middle) Grayscale image of Pvalb stain. (Right) Merged image of GFP (green) and Pvalb (red) stains. Magenta arrows indicate three examples of putative Pvalb+ cells. Note that red and green somata mostly overlap. Scale bars are 50 microns. **DOI:** http://dx.doi.org/10.7554/eLife.18383.004

**Figure 1—figure supplement 2.**
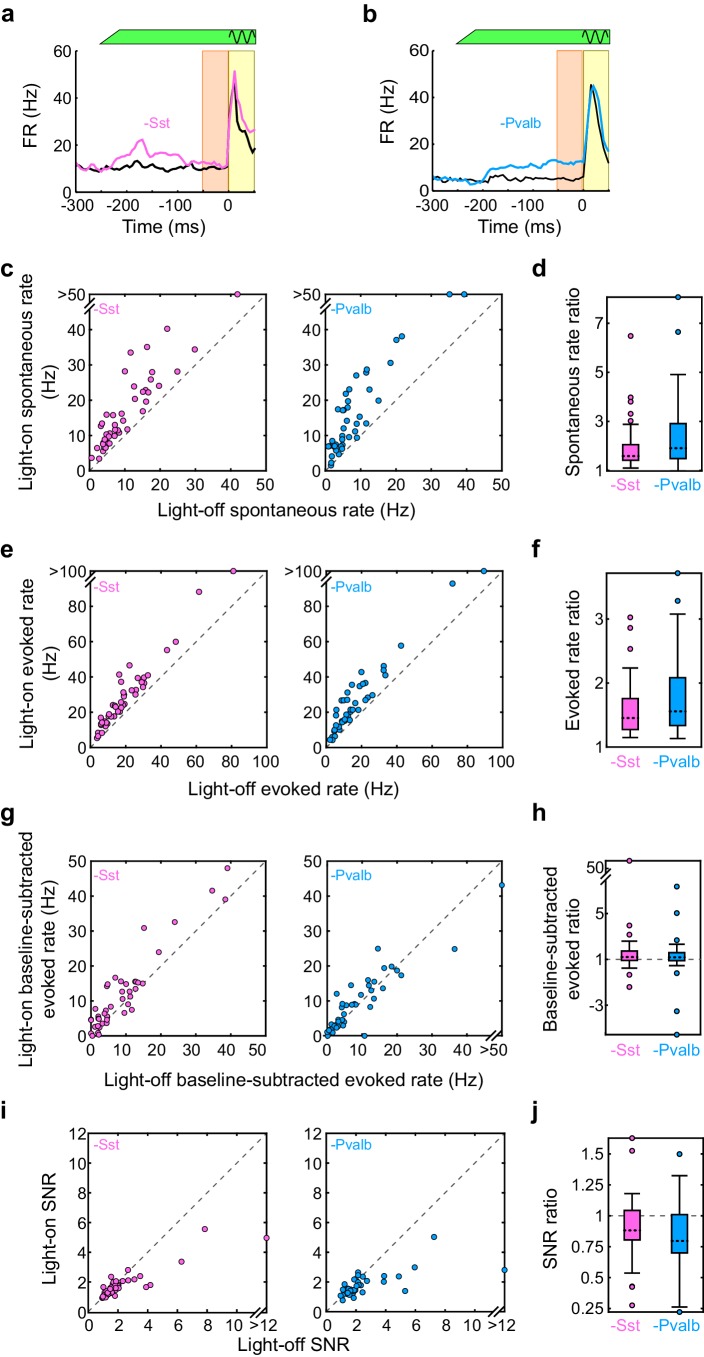
Interneuron inactivation increases firing rates and decreases signal to noise ratio. (**a**) An example unit’s mean firing rate (FR) ± SEMs relative to sound onset, without (black) and with (pink) inactivation of Sst+ interneurons. The black sine wave represents the duration of the sound, the green bar represents the duration and power of the light, the orange bar indicates the 50 ms time period used to calculate spontaneous FR, and the yellow bar indicates the 50 ms response period used to calculate tone-evoked FR. (**b**) Same as (**a**), for an example unit recorded from an Arch/Pvalb mouse without (black) and with (light blue) inactivation of Pvalb+ interneurons. (**c**) The spontaneous FR with versus without light for each tuned unit whose sound-evoked FR was increased by light. Left: Arch/Sst, n = 44 units; right: Arch/Pvalb, n = 41 units. (**d**) Spontaneous rate ratio (defined as light-on FR divided by light-off FR) for the units in (**c**). Inactivation of Sst+ interneurons increases the spontaneous rate ratio (signrank p=7.6 × 10^−9^), as does inactivation of Pvalb+ interneurons (signrank p=2.4 × 10^−8^), but these ratios are not different from each other (ranksum p=0.054). (**e**) Same as (**c**), but for sound-evoked FRs (calculated as average spikes/second in the 50 ms after response onset). (**f**) Sound-evoked rate ratio for the units in (**e**). Inactivation of Sst+ interneurons increases the sound-evoked rate ratio (signrank p=7.5 × 10^−9^), as does inactivation of Pvalb+ interneurons (signrank p=2.2 × 10^−8^), but these ratios are not different from each other (ranksum p=0.26). (**g**) Same as (**c**), but for baseline-subtracted sound-evoked FRs (defined as evoked FR minus spontaneous FR). (**h**) Baseline-subtracted sound-evoked ratio for the units in (**g**). Inactivation of Sst+ interneurons increases the baseline-subtracted sound-evoked ratio (signrank p=8.0 × 10^−7^), as does inactivation of Pvalb+ interneurons (signrank p=5.2 × 10^−6^), but these ratios are not different from each other (ranksum p=0.77). (**i**) Same as (**c**), but for signal to noise ratio (SNR: defined as sound-evoked FR divided by spontaneous FR). (**j**) SNR ratio for the units in (**i**). Inactivation of Sst+ interneurons decreases SNR ratio (signrank p=2.6 × 10^−10^), as does inactivation of Pvalb+ interneurons (signrank p=1.9 × 10^−8^), but these ratios are not different from each other (ranksum p=0.081). **DOI:** http://dx.doi.org/10.7554/eLife.18383.005

### Interpreting optogenetic manipulations in a linear framework

**Figure 2.**
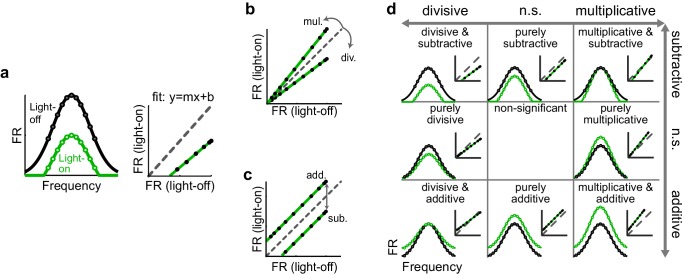
Identifying multiplicative/divisive and additive/subtractive changes in frequency tuning curves with linear regression. (**a**) (Left) Schematic of a neuron’s FTC in the light-off (black) and light-on (green) conditions. (Right) Linear regression of light-off versus light-on firing rates (FRs) (measurements: black; fit: green; unity line: dashed gray). (**b**) Multiplicative/divisive changes produce significant slopes (≠ 1). (**c**) Additive/subtractive changes produce significant intercepts (≠ 0). (**d**) Table showing all combinations of linear transformations of frequency tuning curves and their associated linear fits. **DOI:** http://dx.doi.org/10.7554/eLife.18383.006

To characterize how optogenetically altering inhibition affects a neuron’s tuning curve, we employed a common framework that models the effect as a linear transformation, comprised of scaling (multiplicative/divisive) and shifting (additive/subtractive) components. We calculated these components by fitting a straight line to the average firing rates on trials with (green) versus without (black) altered inhibition (Figure 2a). In the context of optogenetically manipulating interneurons in the auditory cortex, comparing a neuron’s FTC in the light-on versus light-off conditions would be expected to reveal whether the change in firing in that neuron is well fit by such a model, and, if so, whether and to what extent the transformation is multiplicative/divisive (Figure 2b), additive/subtractive (Figure 2c), or a combination (Figure 2d). The slope of the best-fit line, if significantly different from unity, reveals the magnitude of the multiplicative/divisive component of the transformation (Figure 2b), while the y-intercept of the best-fit line, if significantly different from zero, reveals the magnitude and significance of the additive/subtractive component (Figure 2c). Within this framework, a neuron’s FTC may undergo eight possible types of linear transformations (e.g. significantly divisive but not significantly additive/subtractive, significantly divisive and significantly subtractive, significantly subtractive but not significantly multiplicative/divisive, and so on; Figure 2d); alternatively, neither the scaling nor shifting components may be significant at all.

### Optogenetic inactivation produces cell type specific effects on gain and tuning

**Figure 3.**
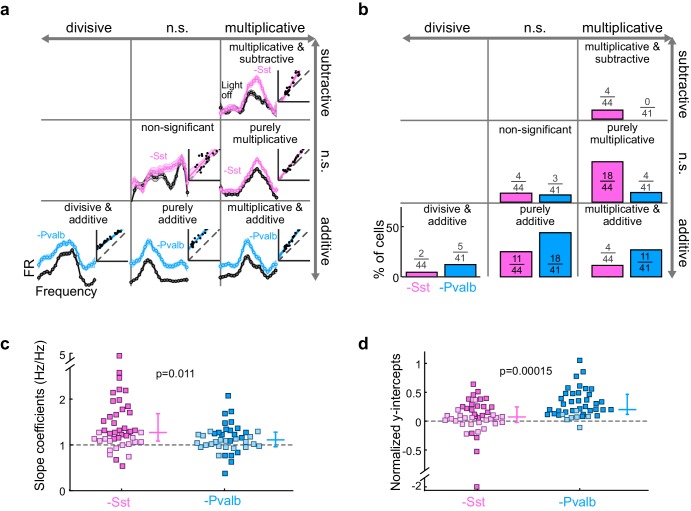
Optogenetically inactivating Sst+ interneurons, compared to inactivating Pvalb+ interneurons, produces different linear transformations of FTCs. (**a**) FTCs (mean ± SEMs) of representative units demonstrating all the combinations of linear transformations observed with inactivation of either Sst+ cells (pink) or Pvalb+ cells (light blue). (**b**) Fraction of units that showed each kind of linear transformation with inactivation of Sst+ cells (pink) and inactivation of Pvalb+ cells (light blue). These proportions are significantly different between groups (Arch/Sst: n = 44 units from 12 mice; Arch/Pvalb: n = 41 units from 11 mice; Fisher’s exact test p=1.5 × 10^−3^). (**c**) Population best-fit slope coefficients with inactivation of Sst+ cells (pink) and inactivation of Pvalb+ cells (light blue). Slopes were significantly different between groups (rank-sum p=0.01). Dark/light squares indicate units for which the slope was/was not significantly different from 1, respectively. Lines indicate population medians and lower/upper quartiles. (**d**) Population best-fit y-intercepts, normalized by maximum firing rate, with inactivation of Sst+ cells (pink) and inactivation of Pvalb+ cells (light blue). Y-intercepts were significantly different between groups (rank-sum p=1.5 × 10^−4^). Dark/light squares indicate units for which the y-intercept was/was not significantly different from 0, respectively. Lines indicate population medians and lower/upper quartiles. **DOI:** http://dx.doi.org/10.7554/eLife.18383.007

What does inactivating Sst+ and Pvalb+ cells lead us to conclude about their specific contributions to the gain (multiplication/division) or tuning (addition/subtraction) of tone responses? We applied the linear regression described above to the 44 units recorded from Arch/Sst and 41 units from Arch/Pvalb mice whose responses were tuned for frequency and significantly increased by light (see Materials and methods). This included both narrow-spiking and broad-spiking units (defined by a trough-to-peak duration of less than or greater than 450 μs, respectively; see Materials and methods). Most units showed predominantly linear changes in their FTC with decreased inhibition (Arch/Sst: median R^2^ = 0.81; Arch/Pvalb: median R^2^ = 0.78; Figure 3—figure supplement 1). Inactivation of Sst+ cells produced a variety of linear transformations (Figure 3a, pink examples), as did inactivation of Pvalb+ cells (Figure 3a, light blue examples). However, the proportions of linear transformation types produced by inactivation of Sst+ cells were significantly different from those produced by inactivation of Pvalb+ cells (Fisher’s exact test p=0.0015; Figure 3b). To quantitatively compare the effects of inactivating Sst+ or Pvalb+ cells on response gain (i.e., the magnitude of the multiplicative component), we compared the distributions of the slope values obtained from the linear regression analyses (Figure 3c) and found that inactivating Sst+ cells had a significantly larger effect on the response gain than inactivating Pvalb+ cells (rank-sum p=0.01). Furthermore, inactivating Pvalb+ cells had a significantly larger effect on the magnitude of the additive component compared to inactivating Sst+ cells (rank-sum p=1.5 × 10^−4^; Figure 3d). These differences between the effects of inactivating Sst+ versus Pvalb+ cells were more prominent among broad-spiking units than narrow-spiking units (Figure 3—figure supplement 2). Interestingly, this larger additive effect produced by inactivating Pvalb+ cells was not entirely due to increases in spontaneous activity, as performing linear regression on baseline-subtracted responses (defined as sound-evoked FR minus spontaneous FR; Figure 3—figure supplement 3a,b) revealed similar differences in both the slopes and y-intercepts (Figure 3—figure supplement 3c,d), implying that the additive effect produced by inactivation of Pvalb+ cells must include a substantial contribution from sound-evoked responses that are not frequency selective.

**Figure 3—figure supplement 1.**
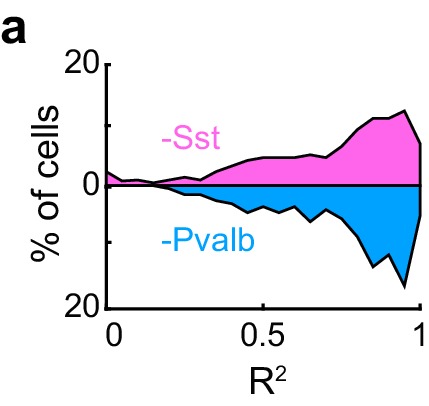
Effects of inactivating Sst+ or Pvalb+ cells are mainly linear. (**a**) Distribution of the R^2^ values from the linear regression analysis from units with activation of Sst+ cells (pink) and activation of Pvalb+ cells (light blue). High median R^2^ values demonstrate that in most units the effects of decreased inhibition are fit well by a linear framework (ChR2/Sst: median R^2^ = 0.81; ChR2/Pvalb: median R^2^ = 0.80). **DOI:** http://dx.doi.org/10.7554/eLife.18383.008

**Figure 3—figure supplement 2.**
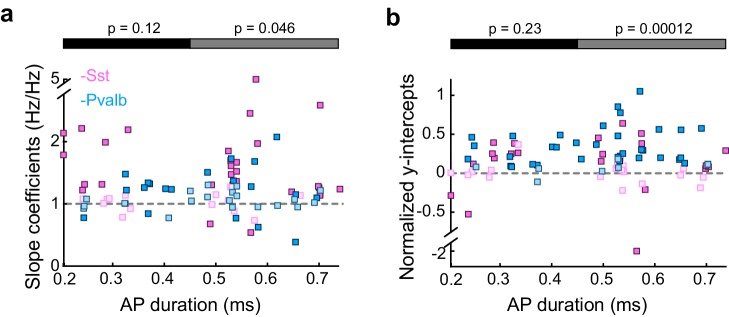
Differences between inactivating Sst+ or Pvalb+ cells are present among broad-spiking, but not narrow-spiking, units. (**a**) Best-fit slope coefficients for each unit as a function of action potential (AP) duration (trough-to-peak duration). Among the units with narrow-spiking waveforms (≤450 μs) whose activity was increased by light (Arch/Sst: n = 15; Arch/Pvalb: n = 15), Sst+ cell inactivation (pink) and Pvalb+ cell inactivation (light blue) produced slopes that were not significantly different (ranksum p=0.12). However, among the units with broad-spiking waveforms (>450 μs) whose activity was increased by light (Arch/Sst: n = 29; Arch/Pvalb: n = 26), Sst+ cell inactivation produced significantly larger slopes (rank-sum p=0.046) than Pvalb+ cell inactivation. Darker squares represent units for which the slope was significant. (**b**) Normalized best-fit y-intercepts for each unit as a function of change in sound-evoked firing rate. Among the units with narrow-spiking waveforms (≤450 μs) whose activity was increased by light (Arch/Sst: n =15; Arch/Pvalb: n = 15), Sst+ cell inactivation (pink) and Pvalb+ cell inactivation (light blue) produced y-intercepts that were not significantly different (rank-sum p=0.23). However, among the units with broad-spiking waveforms (>450 μs) whose activity was increased by light (Arch/Sst: n = 29; Arch/Pvalb: n = 26), Pvalb+ cell inactivation produced larger y-intercepts (rank-sum p=0.00012) than Sst+ cell inactivation. Darker squares represent units for which the normalized y-intercept was significant. **DOI:** http://dx.doi.org/10.7554/eLife.18383.009

**Figure 3—figure supplement 3.**
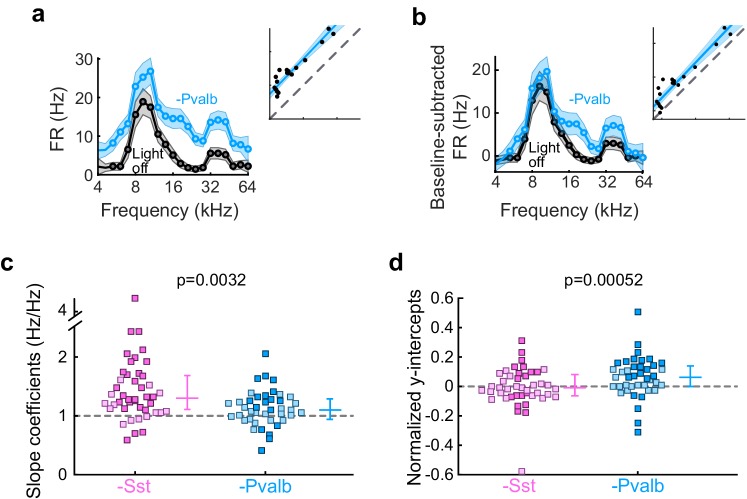
Effects of inactivating Sst+ or Pvalb+ cells are not explained entirely by increases in spontaneous activity. (**a**) An example unit’s FTC (mean ± SEMs) with (light blue) and without (black) inactivation of Pvalb+ cells. Inset shows the linear regression for light-off versus light-on firing rates (FRs) (measured FRs: black dots; fit: light blue line; confidence intervals: light blue shading; unity line: dashed gray line). (**b**) Baseline-subtracted FTCs from the same unit in (**a**). Note that the additive component (y-intercept) is still significant after baseline-subtraction, albeit smaller. (**c**) Best-fit slope coefficients from baseline-subtracted units with inactivation of Sst+ cells (pink) and inactivation of Pvalb+ cells (light blue) were significantly different from each other (rank-sum p=0.0032). Dark/light squares indicate units for which the slope was/was not significantly different from 0, respectively. Lines indicate population medians and lower/upper quartiles. (**d**) Normalized best-fit y-intercepts from baseline-subtracted units with inactivation of Sst+ cells (pink) and inactivation of Pvalb+ cells (light blue) were significantly different from each other (rank-sum, p=0.00052). Dark/light squares indicate units for which the y-intercept was/was not significantly different from 0, respectively. Lines indicate population medians and lower/upper quartiles. **DOI:** http://dx.doi.org/10.7554/eLife.18383.010

**Figure 3—figure supplement 4.**
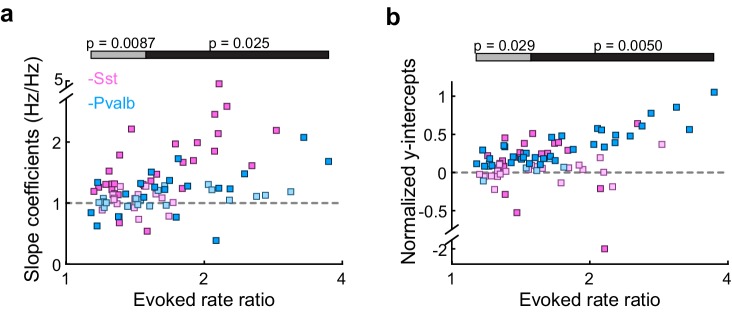
Differences between inactivating Sst+ or Pvalb+ cells are present even in unit populations whose firing rates are weakly affected by interneuron inactivation, as well as in strongly affected unit populations. (**a**) Best-fit slope coefficients for each unit as a function of change in sound-evoked firing rate between the light-on and light-off conditions. Among the half of the units that were more weakly affected by interneuron inactivation (Arch/Sst: n = 25, pink; Arch/Pvalb: n = 18, light blue), Pvalb+ cell inactivation produced smaller slopes than Sst+ cell inactivation (rank-sum p=0.0087). Among the half of the units that were more strongly affected by interneuron inactivation (Arch/Sst: n = 19; Arch/Pvalb: n = 23), Pvalb+ cell inactivation produced smaller slopes than Sst+ cell inactivation (rank-sum p=0.025). Darker squares represent units for which the slope was significant. (**b**) Normalized best-fit y-intercepts for each unit as a function of change in sound-evoked firing rate. Among the half of the units that were more weakly affected by interneuron inactivation (Arch/Sst: n = 25, pink; Arch/Pvalb: n = 18, light blue), Pvalb+ cell inactivation produced larger y-intercepts than Sst+ cell inactivation (rank-sum p=0. 029). Among the half of the units that were more strongly affected by interneuron inactivation (Arch/Sst: n = 19; Arch/Pvalb: n = 23), Pvalb+ cell inactivation produced larger y-intercepts than Sst+ cell inactivation (rank-sum p=0.005). Darker squares represent units for which the y-intercept was significant. **DOI:** http://dx.doi.org/10.7554/eLife.18383.011

**Figure 3—figure supplement 5.**
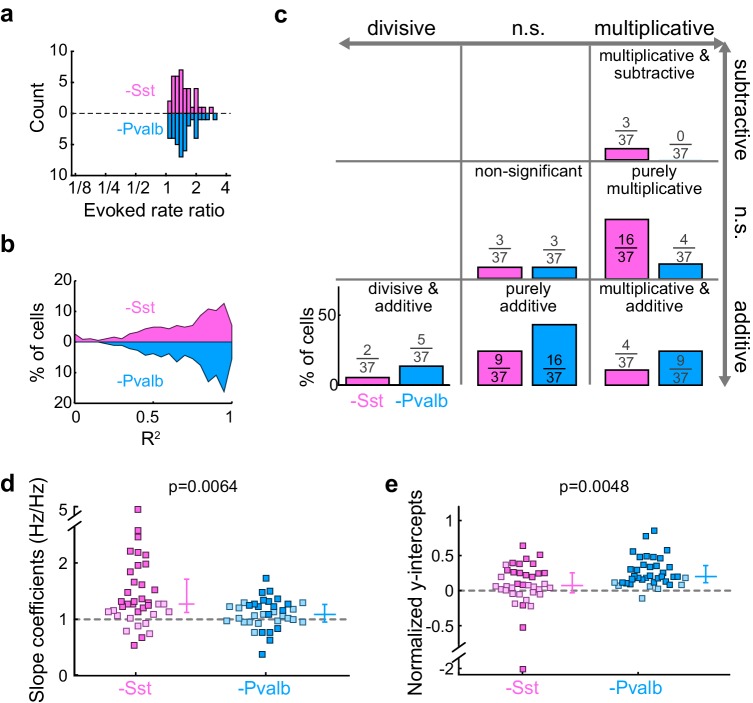
Inactivation of Sst+ cells, compared to inactivation of Pvalb+ cells, produces different proportions of linear effects, even after matching unit populations based on firing rate ratios. (**a**) The linear regression analysis is limited to units from Arch/Sst mice and Arch/Pvalb mice that have similar sound-evoked firing rate ratios (Arch/Sst: n = 37 units from 12 mice; Arch/Pvalb: n = 37 units from 11 mice). (**b**) Distribution of the R^2^ values from the linear regression analysis from ratio-matched units with inactivation of Sst+ cells (pink) and inactivation of Pvalb+ cells (light blue). High median R^2^ values demonstrate that most ratio-matched units are fit well by a linear framework (Arch/Sst: median R^2^ = 0.78; Arch/Pvalb: median R^2^ = 0.87) (**c**) Fraction of units that showed each kind of linear transformation with inactivation of Sst+ cells (pink) or inactivation of Pvalb+ cells (light blue) were significantly different from each other (Arch/Sst: n = 37 units; Arch/Pvalb: n = 37 units; Fisher’s exact test p=0.0058). (**d**) Best-fit slope coefficients from ratio-matched units with inactivation of Sst+ cells (pink) and inactivation of Pvalb+ cells (light blue) were significantly different from each other (rank-sum p=0.0064). Dark/light squares indicate units for which the slope was/was not significantly different from 0, respectively. Lines indicate population medians and lower/upper quartiles. (**e**) Normalized best-fit y-intercepts from ratio-matched units with inactivation of Sst+ cells (pink) and inactivation of Pvalb+ cells (light blue) were significantly different from each other (rank-sum, p=0.0048). Dark/light squares indicate units for which the y-intercept was/was not significantly different from 0, respectively. Lines indicate population medians and lower/upper quartiles. **DOI:** http://dx.doi.org/10.7554/eLife.18383.012

Note that, all other factors being equal, both the slope and y-intercept of the linear regression (i.e., the degree to which a linear transformation registers as multiplicative or additive), and the chances that either of these coefficients will be statistically significant, will be greater in units whose firing rates are more strongly affected by the light (Figure 3—figure supplement 4). Although we observed that even weakly affected unit populations showed significant differences in both slopes and intercepts (Figure 3—figure supplement 4), we wondered whether these differences might still be explained by an overall greater increase in firing rates when inactivating one interneuron type versus the other. We addressed this potential confound by limiting our population analyses to units with similar increases in firing rates (see Materials and methods; Figure 3—figure supplement 5a). In these matched populations, we again found that the proportions of linear transformations produced by inactivating Sst+ or Pvalb+ cells differed (Fisher’s exact test, p=0.0058; Figure 3—figure supplement 5c), as did the magnitudes of both the multiplicative and additive effects (slopes: rank-sum p=0.0064; Figure 3—figure supplement 5d; y-intercepts: rank-sum p=0.0048; Figure 3—figure supplement 5e).

### Optogenetic activation does not produce cell type specific effects on gain and tuning

**Figure 4.**
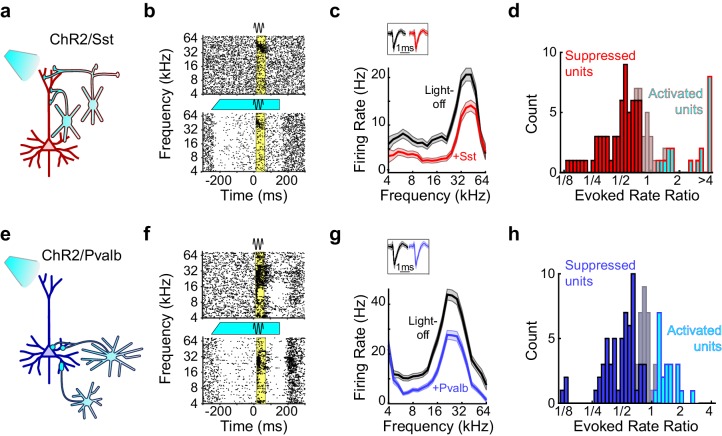
Optogenetic activation of Sst+ or Pvalb+ interneurons. (**a**) Schematic of optogenetic manipulation in ChR2/Sst mice in which blue light directly activates Sst+ cells (cyan cells). (**b**) Rasters of tone-evoked action potentials for a representative suppressed unit without (top) and with (bottom) activation of Sst+ cells. The black sine wave represents the duration of the sound, the cyan bar represents the duration and power of the light, and the yellow bar indicates the response period used to construct FTCs. (**c**) FTCs (mean ± SEMs) derived from tone-evoked firing rates (FRs) without (black) and with (red) activation of Sst+ cells for the representative unit in (**b**). Inset shows unit waveforms on trials without (black) and with (red) activation of Sst+ cells. (**d**) Distribution of the ratio of light-on to light-off FRs during the response period in ChR2/Sst mice for frequency tuned units. Red bars indicate units with significantly suppressed FRs (n = 64 of 97 units), grayish red bars indicate units with no significant change in FR (n = 19 of 97 units), and cyan bars with red outline indicate units with significantly increased FRs (n = 14 of 97 units). (**e–h**) As (**a–b**), but in ChR2/Pvalb mice, in which blue light directly activates Pvalb+ cells (**e**). (**f,g**) show the rasters and FTCs of a representative suppressed unit with and without activation of Pvalb+ cells. In (**h**), blue bars indicate units with significantly suppressed FRs (n = 62 of 103 units), grayish blue bars indicate units with no significant change in FR (n = 20 of 103 units), and cyan bars with blue outline indicate units with significantly increased FRs (n = 19 of 103 units). **DOI:** http://dx.doi.org/10.7554/eLife.18383.013

To perform the complementary experiment, we produced mice in which we could activate Sst+ or Pvalb+ interneurons by crossing Sst- and Pvalb-Cre mouse strains (Taniguchi et al., 2011) with the Ai32 strain, in which expression of ChR2-eYFP is Cre-dependent (Madisen et al., 2012) (hereafter referred to as ChR2/Sst and ChR2/Pvalb mice, respectively; Figure 4—figure supplement 1). In ChR2/Sst mice, somatostatin mostly co-localized with GFP, and in ChR2/Pvalb mice, parvalbumin mostly co-localized with GFP (Figure 4—figure supplement 1). In these mice, blue light had a range of effects on both sound-evoked FRs, spontaneous FRs, and SNRs (Figure 4b,f; Figure 4—figure supplement 2). Of the frequency-tuned units (ChR2/Sst: n = 97 of 163 units; ChR2/Pvalb: n = 103 of 160 units; see Materials and methods), the majority had responses to tones that were significantly reduced by light (ChR2/Sst: n = 64 of 97 units; ChR2/Pvalb: n = 62 of 103 units), a minority had responses that were significantly increased by light (ChR2/Sst: n = 19 of 97 units; ChR2/Pvalb: n = 21 of 103 units), and the remainder of units’ responses showed no significant change (ChR2/Sst: n = 14 of 97 units; ChR2/Pvalb: n = 20 of 103 units). A small fraction of units with no significant change to their tone responses nonetheless showed significant changes to their spontaneous activity (ChR2/Sst: n = 4 of 14; ChR2/Pvalb: n = 3 of 20).

**Figure 4—figure supplement 1.**
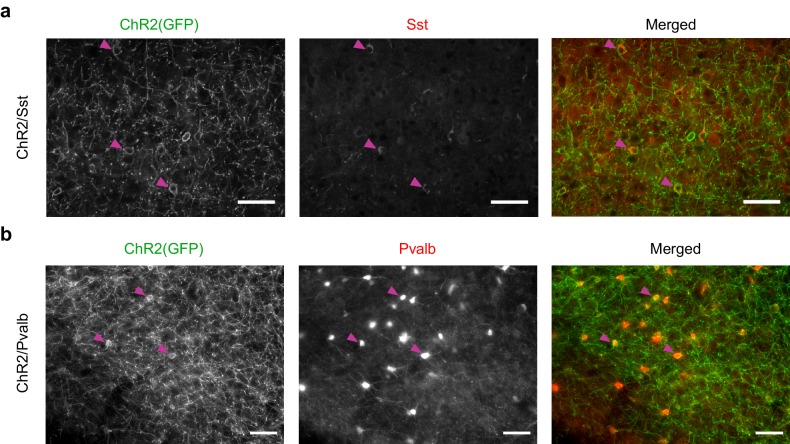
ChR2(GFP) co-localizes with somatostatin in ChR2/Sst mice and with parvalbumin in ChR2/Pvalb mice. (**a**) Immunostaining of a brain slice from a ChR2/Sst mouse. (Left) Grayscale image of GFP stain. (Middle) Grayscale image of somatostatin stain. (Right) Merged image of GFP (green) and somatostatin (red) stains. Magenta arrows indicate three examples of putative Sst+ cells. Note that red and green somata mostly overlap. Scale bars are 50 microns. (**b**) Immunostaining of a brain slice from a ChR2/Pvalb mouse. (Left) Grayscale image of GFP stain. (Middle) Grayscale image of parvalbumin stain. (Right) Merged image of GFP (green) and parvalbumin (red) stains. Magenta arrows indicate three examples of putative Pvalb+ cells. Note that red and green somata mostly overlap. Scale bars are 50 microns. **DOI:** http://dx.doi.org/10.7554/eLife.18383.014

**Figure 4—figure supplement 2.**
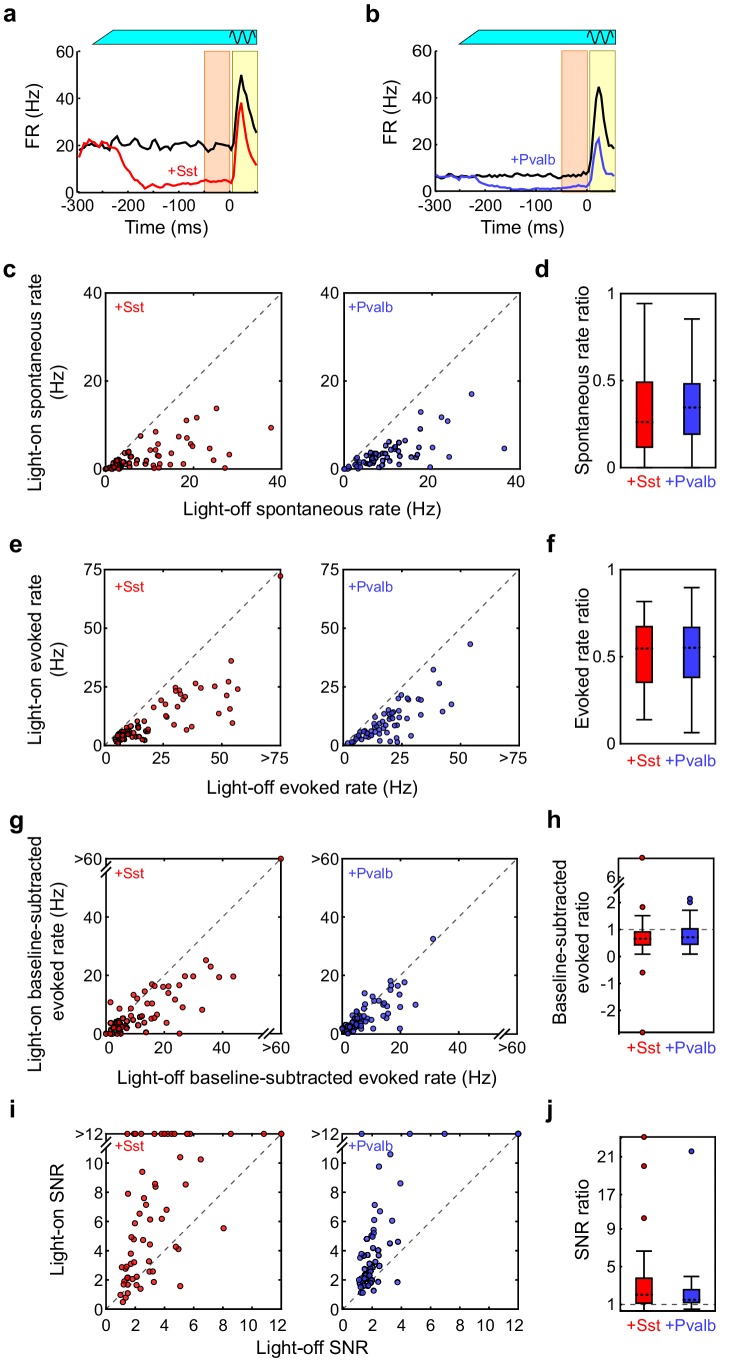
Interneuron activation decreases firing rates and increases signal to noise ratio. (**a**) An example unit’s mean firing rate (FR) ± SEMs relative to sound onset, without (black) and with (red) activation of Sst+ interneurons. The black sine wave represents the duration of the sound, the green bar represents the duration and power of the light, the orange bar indicates the 50 ms time period used to calculate spontaneous FR, and the yellow bar indicates the 50 ms response period used to calculate tone-evoked FR. (**b**) Same as (**a**), but of an example unit recorded from a ChR2/Pvalb mouse without (black) and with (blue) activation of Pvalb+ interneurons. (**c**) The spontaneous firing rate (FR) (calculated as average spikes/second in the 50 ms before the stimulus) with versus without light for each tuned unit whose sound-evoked FR was increased by light. Left: ChR2/Sst, n = 64 units; right: ChR2/Pvalb, n = 62 units. (**d**) Spontaneous rate ratio (defined as light-on FR divided by light-off FR) for the units in (**a**). Activation of Sst+ interneurons decreases the spontaneous rate ratio (signrank p=1.1 × 10^−11^), as does activation of Pvalb+ interneurons (signrank p=5.1 × 10^−11^), but these ratios are not different from each other (ranksum p=0.56). (**e**) Same as (**c**), but for sound-evoked FRs (calculated as average spikes/second in the 50 ms after response onset). (**f**) Sound-evoked rate ratio for the units in (**e**). Activation of Sst+ interneurons decreases the sound-evoked rate ratio (signrank p=3.5 × 10^−12^), as does activation of Pvalb+ interneurons (signrank p=7.6 × 10^−12^), but these ratios are not different from each other (ranksum p=0.76). (**g**) Same as (**c**), but for baseline-subtracted sound-evoked FRs (defined as evoked FR minus spontaneous FR). (**h**) Baseline-subtracted sound-evoked ratio for the units in (**g**). Activation of Sst+ interneurons decreases the baseline-subtracted sound-evoked ratio (signrank p=2.2 × 10^−10^), as does activation of Pvalb+ interneurons (signrank p=7.6 × 10^−12^), but these ratios are not different from each other (ranksum p=0.31). (**i**) Same as (**c**), but for signal to noise ratio (SNR: defined as sound-evoked FR divided by spontaneous FR). (**j**) SNR ratio for the units in (**i**). Activation of Sst+ interneurons increases SNR ratio (signrank p=5.2 × 10^−12^), as does activation of Pvalb+ interneurons (signrank p=1.6 × 10^−11^), but these ratios are not different from each other (ranksum p=0.20). **DOI:** http://dx.doi.org/10.7554/eLife.18383.015

**Figure 5.**
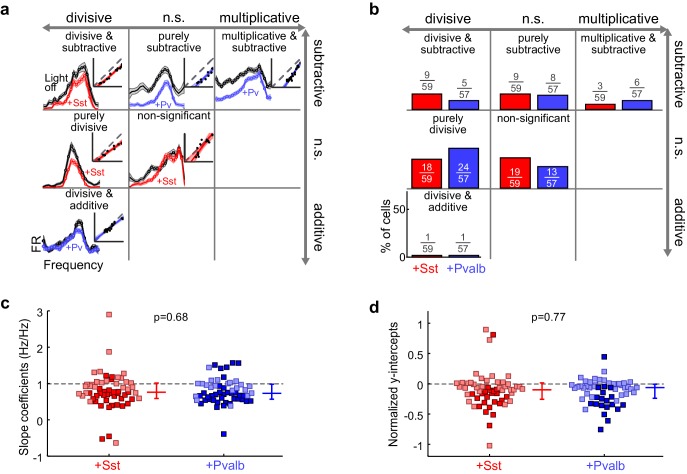
Optogenetically activating Sst+ interneurons, compared to activating Pvalb+ interneurons, produces similar linear transformations of frequency tuning curves. (**a**) FTCs (mean ± SEMs) of representative units demonstrating all the combinations of linear transformations observed with activation of either Sst+ cells (red) or Pvalb+ cells (blue). (**b**) Fraction of units that showed each kind of linear transformation with activation of Sst+ cells (red) and activation of Pvalb+ cells (blue). Distribution of proportions is not significantly different between groups (ChR2/Sst: n = 59 units from 25 mice; ChR2/Pvalb: n = 57 units from 26 mice; Fisher’s exact test p=0.51). (**c**) Best-fit slope coefficients for activation of Sst+ cells (red) and activation of Pvalb+ cells (blue). Slopes were not significantly different between groups (rank-sum p=0.68). Dark/light squares indicate units for which slope was/was not significantly different from 1, respectively. Lines indicate population medians and lower/upper quartiles. (**d**) Best-fit y-intercepts, normalized by maximum firing rate, for activation of Sst+ cells (red) and activation of Pvalb+ cells (blue). Y-intercepts were not significantly different between groups (rank-sum, p=0.77). Dark/light squares indicate units for which y-intercept was/was not significantly different from 0, respectively. Lines indicate population medians and lower/upper quartiles. **DOI:** http://dx.doi.org/10.7554/eLife.18383.016

To evaluate how interneuron activation suppresses auditory cortical responses, we applied the same linear framework to the FTCs of the 59 units from ChR2/Sst and 57 units from ChR2/Pvalb mice whose responses were both tuned for frequency and significantly suppressed by activation of interneurons (Figure 4d,h; Figure 4—figure supplement 2), excluding units that were suppressed by more than 80% (to avoid floor effects), and including both narrow-spiking and broad-spiking units. Among these units, this linear framework explained the effects of light-driven inhibition from Sst+ and Pvalb+ cells well, as evidenced by median R^2^ values around 0.8 (ChR2/Sst: median R^2^ = 0.81; ChR2/Pvalb: median R^2^ = 0.80; Figure 5—figure supplement 1). Activating Sst+ cells, and activating Pvalb+ cells, both produced a variety of types of linear transformation (Figure 5a, red and blue examples, respectively).

**Figure 5—figure supplement 1.**
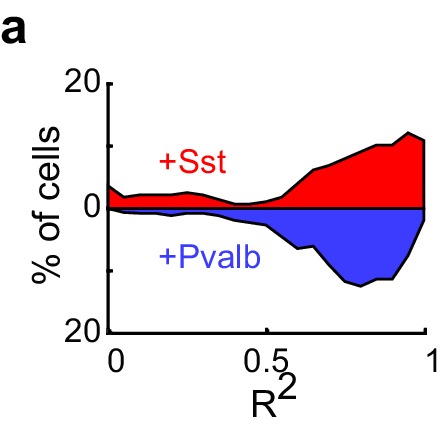
Effects of activating Sst+ or Pvalb+ cells are mainly linear. (**a**) Distribution of the R^2^ values from the linear regression analysis from units with activation of Sst+ cells (red) and activation of Pvalb+ cells (blue). High median R^2^ values demonstrate that response changes in most units are fit well by a linear framework (ChR2/Sst: median R^2^ = 0.81; ChR2/Pvalb: median R^2^ = 0.80). **DOI:** http://dx.doi.org/10.7554/eLife.18383.017

**Figure 5—figure supplement 2.**
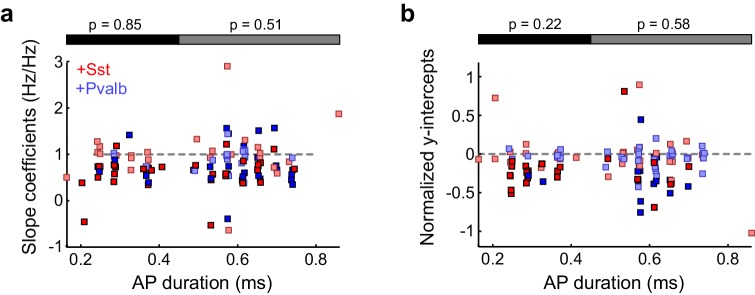
Broad- and narrow-spiking units are similarly affected by interneuron activation. (**a**) Best-fit slope coefficients for each unit as a function of action potential (AP) duration (trough-to-peak duration). Among the units with narrow-spiking waveforms (≤450 μs) whose activity was suppressed by light (ChR2/Sst: n =29; ChR2/Pvalb: n = 12), Sst+ cell activation (red) and Pvalb+ cell activation (blue) produced similar slopes (ranksum p=0.85). Likewise, among the units with broad-spiking waveforms (>450 μs) whose activity was suppressed by light (ChR2/Sst: n = 30; ChR2/Pvalb: n = 45), Sst+ activation and Pvalb+ cell activation produced similar slopes (rank-sum p=0.51). Darker squares represent units for which the slope was significant. (**b**) Normalized best-fit y-intercepts for each unit as a function of change in sound-evoked firing rate. Among the units with narrow-spiking waveforms (≤450 μs) whose activity was suppressed by light (ChR2/Sst: n = 29; ChR2/Pvalb: n = 12), Sst+ cell activation (red) and Pvalb+ cell activation (blue) produced similar y-intercepts (rank-sum p=0.22). Likewise, among the units with broad-spiking waveforms (>450 μs) whose activity was suppressed by light (ChR2/Sst: n = 30; ChR2/Pvalb: n = 45), Sst+ activation and Pvalb+ cell activation produced similar y-intercepts (rank-sum p=0.58). Darker squares represent units for which the normalized y-intercept was significant. **DOI:** http://dx.doi.org/10.7554/eLife.18383.018

**Figure 5—figure supplement 3.**
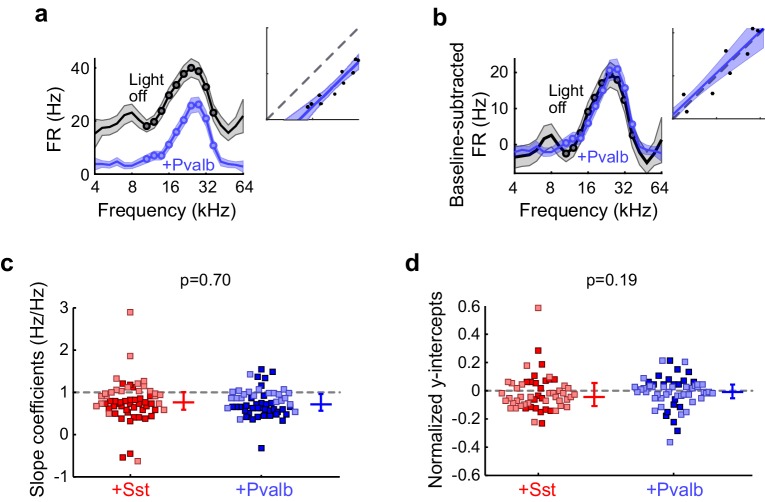
Similar effects of activating Sst+ or Pvalb+ cells remain after baseline subtraction. (**a**) An example unit’s FTC (mean ± SEMs) with (blue) and without (black) activation of Pvalb+ cells. Inset shows the linear regression for light-off versus light-on firing rates (FRs) (measured FRs: black dots; fit: blue line; confidence intervals: blue shading; unity line: dashed gray line). (**b**) Baseline-subtracted FTCs from the same unit in (**a**). Note that the subtractive component (y-intercept) is no longer significant after baseline-subtraction. (**c**) Best-fit slope coefficients from baseline-subtracted units with activation of Sst+ cells (red) and activation of Pvalb+ cells (blue) were similar to each other (rank-sum p=0.70). Dark/light squares indicate units for which slope was/was not significantly different from 0, respectively. Lines indicate population medians and lower/upper quartiles. (**d**) Normalized best-fit y-intercepts from baseline-subtracted units with activation of Sst+ cells (red) and activation of Pvalb+ cells (blue) were similar to each other (rank-sum, p=0.19). Dark/light squares indicate units for which y-intercept was/was not significantly different from 0, respectively. Lines indicate population medians and lower/upper quartiles. **DOI:** http://dx.doi.org/10.7554/eLife.18383.019

**Figure 5—figure supplement 4.**
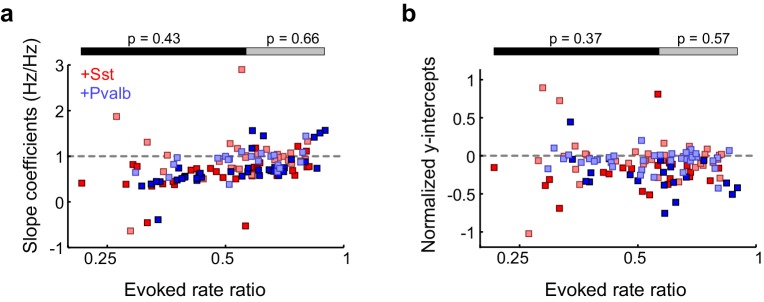
Similarities between activating Sst+ or Pvalb+ cells are present even in unit populations whose firing rates are strongly suppressed, as well as in weakly-suppressed unit populations. (**a**) Slope coefficients for each unit as a function of change in sound-evoked firing rate between the light-on and light-off conditions. Among the half of the units that were more weakly affected by interneuron activation (ChR2/Sst: n = 28, red; ChR2/Pvalb: n = 30, blue), Pvalb+ cell activation produced similar slopes to Sst+ cell activation (rank-sum p=0.66). Among the half of the units that were more strongly affected by interneuron activation (ChR2/Sst: n = 31; ChR2/Pvalb: n = 27), Pvalb+ cell activation produced similar slopes to Sst+ cell activation (rank-sum p=0.43). Darker squares represent units for which the slope was significant. (**b**) Normalized y-intercepts for each unit as a function of change in FR. Among the half of the units that were more weakly affected by interneuron activation (ChR2/Sst: n = 28, red; ChR2/Pvalb: n = 30, blue), Pvalb+ cell activation produced similar y-intercepts to Sst+ cell activation (rank-sum p=0.57). Among the half of the units that were more strongly affected by interneuron activation (ChR2/Sst: n = 31; ChR2/Pvalb: n = 27), Pvalb+ cell activation produced similar y-intercepts to Sst+ cell activation (rank-sum p=0.37). Darker squares represent units for which the y-intercept was significant. **DOI:** http://dx.doi.org/10.7554/eLife.18383.020

**Figure 5—figure supplement 5.**
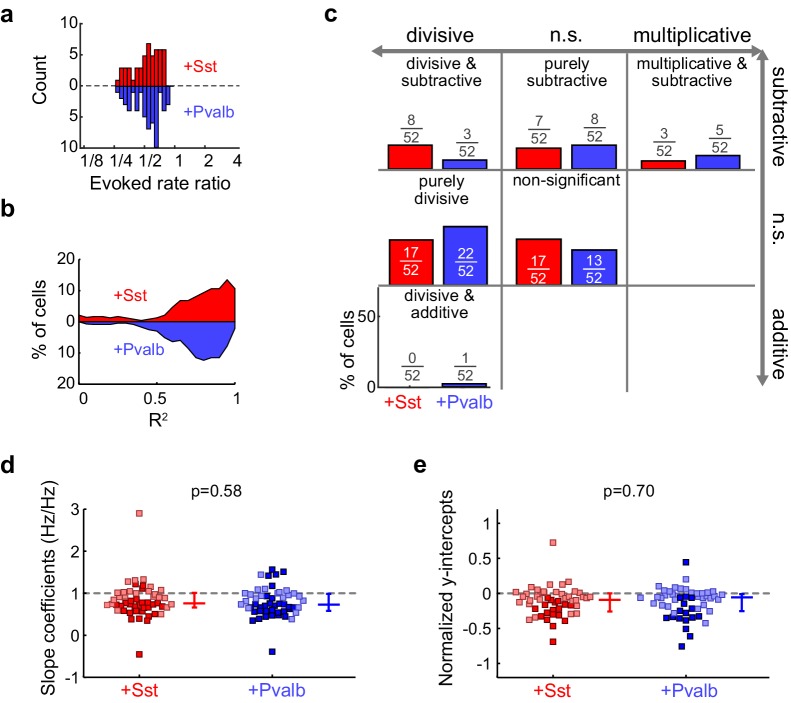
Activation of Sst+ cells, compared to activation of Pvalb+ cells, produces similar linear effects, even after matching unit populations based on firing rate suppression ratios. (**a**) Only units from both populations that have similar firing rate suppression ratios are kept for the analysis (ChR2/Sst: n = 52 units from 24 mice; ChR2/Pvalb: n = 52 units from 25 mice). (**b**) Distribution of the R^2^ values from the linear regression analysis from ratio-matched units with activation of Sst+ cells (red) and activation of Pvalb cells (blue). High median R^2^ values demonstrate that most ratio-matched units are fit well by a linear framework (ChR2/Sst: median R^2^ = 0.84; ChR2/Pvalb: median R^2^ = 0.80). (**c**) Fraction of units that showed each kind of linear transformation with activation of Sst+ cells (red) or activation of Pvalb+ cells (blue). These proportions are not significantly different between groups (ChR2/Sst: n = 52 units; ChR2/Pvalb: n = 52 units; Fisher’s exact test p=0.42). (**d**) Best-fit slope coefficients from ratio-matched units with activation of Sst+ cells (red) and activation of Pvalb+ cells (blue). Slopes were not significantly different between groups (rank-sum p=0.58). Dark/light squares indicate units for which slope was/was not significantly different from 0, respectively. Lines indicate population medians and lower/upper quartiles. (**e**) Normalized best-fit y-intercepts from ratio-matched units with activation of Sst+ cells (red) and activation of Pvalb+ cells (blue). Y-intercepts were not significantly different between groups (rank-sum, p=0.70). Dark/light squares indicate units for which y-intercept was/was not significantly different from 0, respectively. Lines indicate population medians and lower/upper quartiles. **DOI:** http://dx.doi.org/10.7554/eLife.18383.021

**Figure 5—figure supplement 6.**
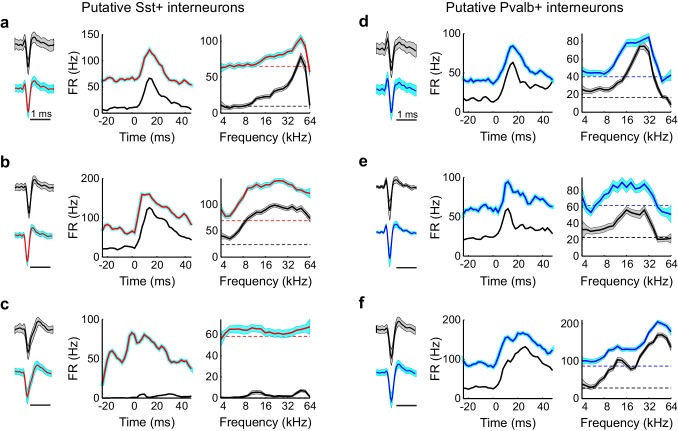
Optogenetic activation of Sst+ and Pvalb+ interneurons increases stimulus-independent activity. (**a**) Left: Waveforms (mean ± SD) from a putative Sst+ interneuron without (black) and with (red line, cyan outline) optogenetic activation. Middle: Peri-stimulus time histogram (mean ± SE) of the putative Sst+ interneuron without (black) and with (red line, cyan outline) optogenetic activation. Time zero indicates sound onset. Right: Frequency tuning curves (mean ± SE) without (black) and with (red line, cyan outline) optogenetic activation. Dotted lines indicate baseline firing rate without (black) and with (red) optogenetic activation. (**b,c**) Same as (**a**) for two other putative interneurons. In (**c**), note that the putative Sst+ interneuron barely increases firing to the stimulus without optogenetic activation; but with optogenetic activation, its firing rate is often above 60 Hz. (**d–f**) Same as (**a–c**) for three putative Pvalb+ interneurons. **DOI:** http://dx.doi.org/10.7554/eLife.18383.022

Counterintuitively, given the observation that inactivation of Sst+ versus Pvalb+ interneurons caused different effects on the gain and tuning of tone responses, the proportions of significant linear transformations observed when activating Sst+ cells and activating Pvalb+ cells were similar to one another (Fisher’s exact test p=0.51; Figure 5b). Furthermore, the distributions of the slope values and the distributions of the y-intercept values obtained from the linear regression analyses were similar (slopes: rank-sum p=0.68; Figure 5c; y-intercepts: rank-sum p=0.77; Figure 5d), consistent with our previous work (Seybold et al., 2015). These similarities were present among broad-spiking units as well as narrow-spiking units (Figure 5—figure supplement 2). Performing linear regression on baseline-subtracted responses (Figure 5—figure supplement 3a,b) also revealed no differences (Figure 5—figure supplement 3c,d). Even among the units with the most suppressed sound-evoked FRs, the effects of Sst+ and Pvalb+ activation were not significantly different from one another (Figure 5—figure supplement 4), suggesting that these similarities were not due to insufficient activation of interneurons. To account for potential differences in the distributions of FR suppression produced by activation of each interneuron population, we again matched the two unit populations by their FR ratios. The proportions of linear transformations produced by interneuron activation remained similar (Fisher’s exact test p=0.42; Figure 5—figure supplement 5c), as did the magnitudes of both the subtractive and divisive effects (slopes: rank-sum p=0.58; Figure 5—figure supplement 5d; y-intercepts: rank-sum p=0.70; Figure 5—figure supplement 5e). In sum, the results of our inactivation experiments would imply that Sst+ and Pvalb+ cells normally support different computations; yet these activation experiments would lead to the conclusion that these neurons do not support different functions.

This may relate to the effects of activation on the interneurons themselves. We identified putative Sst+ (n = 24) and Pvalb+ (n = 27) units by their increased sound-evoked firing rates with light activation (seeMaterials and methods; example putative interneurons: Figure 5—figure supplement 6). These units had median spike trough-to-peak durations of 348 ± 61 μs and 287 ± 41 μs, respectively, were generally spontaneously active before the tone (putative Sst+: FR = 6.3 ± 2.5 Hz; putative Pvalb+: FR = 8.6 ± 4.2 Hz), and increased their firing during the tone (putative Sst+: FR = 12.2 ± 8.5 Hz; putative Pvalb+: FR = 24.8 ± 12.7 Hz). Interestingly, optogenetic activation of putative Sst+ and Pvalb+ interneurons had a larger effect on spontaneous FRs (putative Sst+: FR ratio = 3.9 ± 2.9; putative Pvalb+: FR ratio = 2.5 ± 0.8), than sound-evoked FRs (putative Sst+: FR ratio = 2.9 ± 1.5; putative Pvalb+: FR ratio = 1.5 ± 0.2). Moreover, even putative interneurons that were not generally active without light (sound-evoked rate of less than 5 Hz; putative Sst+: n = 7 units; putative Pvalb+: n = 1 unit) often showed FRs well above 30 Hz during the light (putative Sst+: evoked rate = 35.04 ± 28.3 Hz; putative Pvalb+: evoked rate = 54.5 Hz). Together, these results suggest that activation increases the number of stimulus-independent spikes generated by both interneuron populations, potentially masking normal stimulus-driven function.

### Optogenetic activation and inactivation produce asymmetric effects on information content

**Figure 6.**
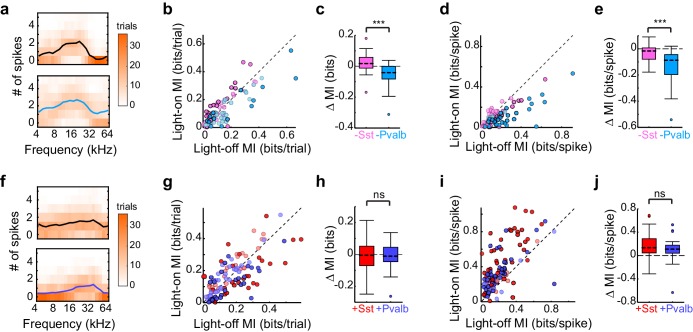
Optogenetically inactivating Sst+ versus Pvalb+ cells produces different effects on mutual information (MI), whereas activating these interneurons produces similar effects on MI. (**a**) Responses of a representative unit recorded in an Arch/Pvalb mouse to tones of different frequencies on trials without (top) and with (bottom) inactivation of Pvalb+ cells (orange heat map: trial counts; line: mean spikes-per-trial). In this unit, information-per-trial between the stimulus and response decreased when Pvalb+ cells were inactivated. (**b**) Information-per-trial with versus without inactivation of Sst+ cells (pink) or Pvalb+ cells (light blue). Darker circles represent units for which the change in information-per-trial was significant (Arch/Sst: n = 44 units from 17 mice; Arch/Pvalb: n = 41 units from 24 mice). (**c**) Box-and-whisker plots of the change in information-per-trial with inactivation of Sst+ cells or Pvalb+ cells. Inactivating Sst+ cells significantly increased information-per-trial (sign-rank p=0.022), inactivating Pvalb+ cells significantly decreased information-per-trial (sign-rank p=1.3 × 10^−4^), and these effects were significantly different from each other (rank-sum p=7.1 × 10^−6^). (**d**) As (**b**) but for information-per-spike, to account for possible differences in the number of spikes available to represent information. (**e**) As (**c**) but for information-per-spike. Inactivating Sst+ cells (sign-rank p=3.5 × 10^−3^) and inactivating Pvalb+ cells (sign-rank p=4.1 × 10^−8^) both significantly decreased information-per-spike, but these effects were significantly different from each other (rank-sum p=1.2 × 10^−5^), indicating that differences in information-per-trial were not due to differences in the number of spikes available to represent information. (**f**) As (**a**) but from a unit recorded in a ChR2/Pvalb mouse. In this unit, information-per-trial between the stimulus and response increased when Pvalb+ cells were activated. (**g**) Information-per-trial with versus without activation of Sst+ cells (red) or Pvalb+ cells (blue). Darker circles represent units for which the change in information-per-trial was significant (ChR2/Sst: n = 59 units from 25 mice; ChR2/Pvalb: n = 57 units from 27 mice). (**h**) Box-and-whisker plots of the change in information-per-trial with activation of Sst+ cells (red) or Pvalb+ cells (blue). Neither activation of Sst+ cells (sign-rank p=0.53) nor activation of Pvalb+ cells (sign-rank p=0.63) changed information-per-trial, and their activation did not produce significantly different effects on information-per-trial (rank-sum p=0.85). (**i**) As (**g**) but for information-per-spike. (**j**) As (**h**) but for information-per-spike. Activating Sst+ cells (sign-rank p=3.9 × 10^−9^) and activating Pvalb+ cells (sign-rank p =1.5 × 10^−8^) both significantly increased the information-per-spike, but these effects were not significantly different from each other (rank-sum p=0.20). **DOI:** http://dx.doi.org/10.7554/eLife.18383.023

This apparent incongruity between the effects of inactivation and activation of interneurons on the gain and tuning of frequency tuning curves prompted us to ask whether we would observe a similar discrepancy in other aspects of sensory processing. Synaptic inhibition can enhance the transfer of information regarding stimulus identity in multiple ways: for instance, rapidly fluctuating inhibition might improve information transfer by linearizing responses (Hasenstaub et al., 2005; Sohal et al., 2009), slower feedforward inhibition might optimize information transfer by adapting the dynamic range of the neuron to the dynamic range of its inputs (Tan et al., 2008), while changes in tonic inhibition might selectively delete irrelevant spikes or change the number of spikes available to represent information (Duguid et al., 2012). These inhibitory dynamics and computational features have been differentially linked to Sst+ or Pvalb+ interneurons (Cardin et al., 2009; Hasenstaub et al., 2005; Sohal et al., 2009; Tan et al., 2008; Wehr and Zador, 2003). To test whether optogenetically inactivating and activating these interneurons also produced discrepant effects on information transfer through cortical neurons, we calculated the per-trial mutual information (in bits/trial; see Materials and methods) between the stimulus and firing rate output, in both the light-off and light-on conditions, for each unit analyzed previously (Figure 6a,f, example units). The effects of inactivating Sst+ cells on information-per-trial were significantly different from the effects of inactivating Pvalb+ cells (rank-sum p=7.1 × 10^−6^; Figure 6b,c): inactivating Sst+ cells significantly increased information-per-trial (sign-rank p=0.022), while inactivating Pvalb+ cells significantly decreased it (sign-rank p=1.3 × 10^−4^). Are these different effects on information transfer produced by inactivation of Sst+ versus Pvalb+ cells simply related to overall changes in the number of spikes available to represent information? To address this, we calculated firing-rate-normalized mutual information (i.e., the information about the stimulus carried by each spike). The effects on information-per-spike of inactivating Sst+ cells versus Pvalb+ cells were significantly different (rank-sum p=1.2 × 10^−5^), inconsistent with the notion that the observed differences in information transfer were due to differences in firing rate modulation (Figure 6d,e).

In contrast, activating Sst+ cells and activating Pvalb+ cells did not differentially impact information-per-trial (rank-sum p=0.85): indeed, neither activation of Sst+ cells nor activation of Pvalb+ cells significantly changed information-per-trial (ChR2/Sst: sign-rank p=0.53; ChR2/Pvalb: sign-rank p=0.63; Figure 6g,h). This lack of differential impact on information-per-trial was likely not due to insufficient activation of interneurons because even the most strongly suppressed units did not exhibit significant differences (ChR2/Sst and ChR2/Pvalb: rank-sum p=0.50). These results are consistent with our previous work, which found no differences between activation of Sst+ or Pvalb+ interneurons on tuning bandwidths (Seybold et al., 2015). Additionally, the incongruity between inactivation and activation (specifically, that inactivation does reveal significant differences in interneuron regulation of information transfer, whereas activation does not) was apparent even after matching units based on the amount that they their firing rates were suppressed or enhanced (Arch/Sst and Arch/Pvalb: rank-sum p=1.7 × 10^-−5^; ChR2/Sst and ChR2/Pvalb: rank-sum p=0.65).

### Inactivation and activation produce asymmetric effects in a convergent network

**Figure 7.**
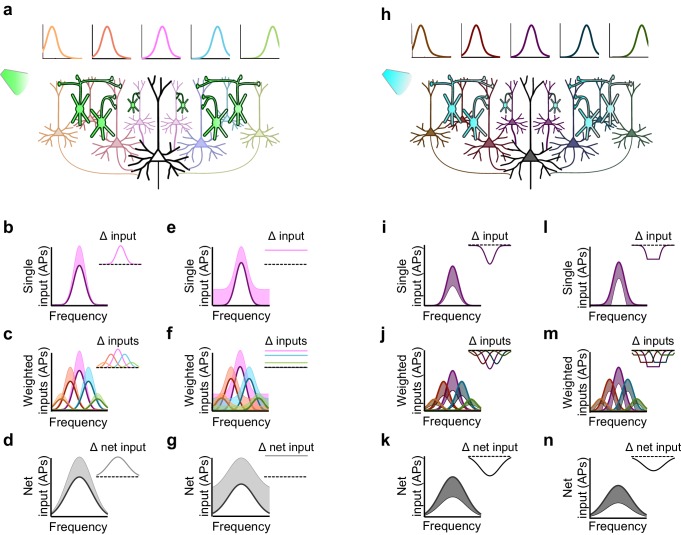
Inactivation versus activation of interneurons can have asymmetrical effects on response gain in a simple network model. (**a**) A population of neurons tuned to different frequencies (light colored cells) provides input to a downstream neuron (large black neuron) in a weighted fashion, such that neurons with more similar tuning to that of the downstream neuron are more strongly connected to it (indicated by color and axon thickness). Interneurons that inhibit these input neurons (green cells) are inactivated, which increases the activity of the input neurons. Number of input neurons: 101; maximum connection weight: 0.2; connection weight falloff: Gaussian with 2SD; input neuron threshold: 0. (**b**) The frequency tuning curve (FTC) of a single neuron in the input layer (light-off: purple curve) is multiplicatively increased by interneuron inactivation (light-on: light purple shading). Inset shows the change in firing. Scaling factor: 1.5. (**c**) As (**b**), but with many weighted inputs. (**d**) The net input to the downstream neuron (i.e., the sum of the weighted inputs) without (black curve) and with (light gray shading) multiplicative scaling of the inputs shows multiplicative scaling. Inset shows the change in net input to the downstream neuron. (**e–g**) As (**b–d**), but demonstrating additive increases in the FTC of a single input (**e**), many weighted inputs (**f**), and the net input to the downstream neuron (**g**). Note that when inactivating interneurons, the linear effects of multiplication and addition at the single-input level are preserved in the net input of the downstream neuron. Shifting factor: 0.15. (**h**) As (**a**), but with activation of interneurons (blue cells), which suppresses the activity of the input neurons (dark colored cells). (**i–k**) As (**b–d**), but demonstrating the effects of division in a single input (**i**), many weighted inputs (**j**), and the net input to the downstream neuron (**k**). Scaling factor: 0.5. (**l–n**) As (**b–d**), but demonstrating the effects of subtraction in the FTC of a single input (**l**), many weighted inputs (**m**), and the net input to the downstream neuron (**n**). Note that the change in net input in the downstream neuron caused by subtraction of inputs appears similar to the change caused by division of inputs. This is due to responses of single input neurons that are suppressed below threshold with subtractive inhibition, causing smaller changes in firing at the edges of the tuning curve than at the center. Shifting factor: -0.15. **DOI:** http://dx.doi.org/10.7554/eLife.18383.024

The ostensible functional equivalencies between Sst+ and Pvalb+ interneurons we observed with optogenetic activation, yet functional differences produced by their inactivation, is paradoxical: how can these supposedly complementary manipulations produce such inconsistent effects? We modeled the effects of inactivating and activating interneuron populations in a densely connected network. We reasoned that not only will optogenetic manipulations of interneurons exert first-order effects on a given neuron, through direct inhibition or removal of inhibition, but they will also yield second-order effects, by altering the network-wide activity of the neuron’s inputs. Consider an example in which one downstream neuron receives inputs from several upstream ('input-layer') neurons (Figure 7a,h). Each input neuron shows evoked responses that are tuned to particular frequencies, superimposed on a modest baseline level of activity, as is often the case in awake states (rev. [Haider and McCormick, 2009]). Neurons with more similar tuning to that of the downstream neuron are more strongly connected to it. The net drive to the downstream neuron is the sum of these weighted inputs. How will multiplicative, additive, divisive, or subtractive modulation propagate through this simple layered network?

Multiplicatively modulating the firing of the upstream layer (Figure 7b) linearly increases the firing rate of each of the weighted inputs (Figure 7c), which sum in the downstream neuron to produce multiplication of its net drive (Figure 7d). Thus multiplication of the input neurons’ responses produces the strongest increase in firing near the center of the downstream neuron’s tuning curve, where stimuli evoke the strongest responses (Figure 7d). Similarly, additively modulating a single input (Figure 7e) produces a constant increase in the firing rate of each weighted input (Figure 7f), which, summed in the downstream neuron, leads to a constant (additive) increase in its net drive across all stimulus frequencies (Figure 7g). Thus, inactivation of interneurons preserves the single-cell effects of multiplication and addition at the network level in this simple model.

**Figure 8.**
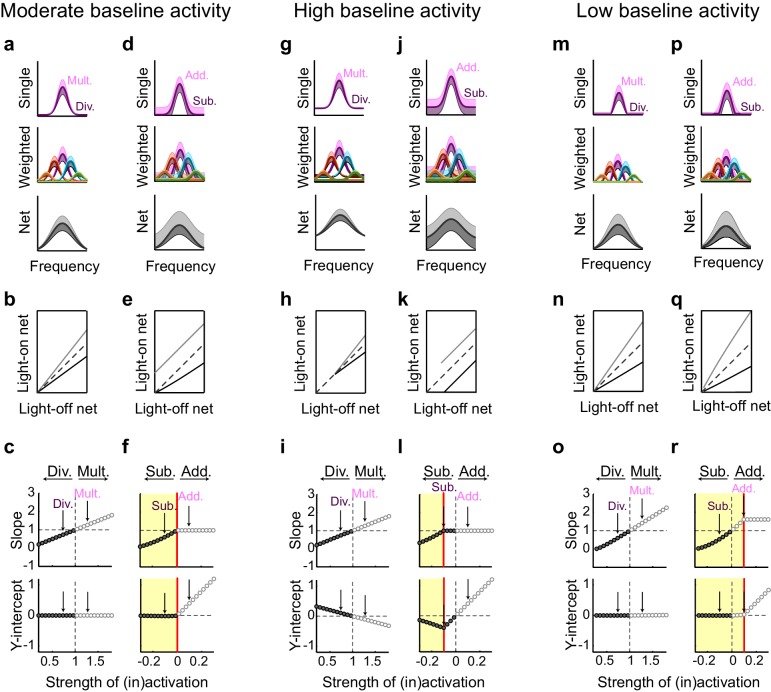
Small changes in key parameters determine whether interneuron manipulations will produce consistent or paradoxical conclusions regarding interneuron function. (**a**) Top row: In a network with moderate baseline activity (threshold = 0), the frequency tuning curve (FTC) of a single input (light-off: medium purple curve) undergoes multiplication (light-purple shading) or division (dark-purple shading). Middle row: The FTCs of many inputs (light-off: medium colored curves), weighted by a Gaussian connectivity function, undergo multiplication (light colored shading) or division (dark colored shading). Bottom row: These weighted inputs sum to produce the downstream neuron’s net input. (**b**) The net input to the downstream neuron without light versus with light (multiplication: light gray line; division: black line). The best-fit slopes and y-intercepts of these measurements represent the magnitudes of the multiplicative/divisive and additive/subtractive effects, respectively, on the downstream neuron’s net input. (**c**) The best-fit slopes and y-intercepts from regressions on the measurements in (**b**) as a function of the strength of (in)activation (i.e., the magnitudes of the linear effects on the downstream neuron’s net input as a function of the magnitudes of the linear effects on the input neurons’ FTCs). Arrows indicate the strengths of interneuron (in)activation represented in (**a**). Under these conditions, interneuron inactivation and activation result in multiplication and division, respectively, of the downstream neuron’s net input. (**d–f**) Same as (**a–c**), except the FTCs of the inputs undergo either addition (light-purple shading) or subtraction (dark-purple shading). Yellow highlighted area in (**f**) indicates the range of interneuron manipulation that interacts with threshold nonlinearities. Interneuron activation interacts with threshold nonlinearities to transform subtraction of the inputs’ FTCs into division of the downstream neuron’s net input, resulting in asymmetric results between activation and inactivation. (**g–l**) Same as (**a–f**), after changing one model parameter: increasing the baseline activity level of the model neurons (threshold = -0.1). Increased baseline activity levels require stronger interneuron activation to cause subtraction of the inputs to appear division-like in the downstream neuron’s net input. This increases the range over which activation/inactivation will produce internally consistent conclusions. (**m–r**) Same as (**a–f**), after changing one model parameter: decreasing the baseline activity level of the model neurons (threshold = 0.1). The weaker responses in (**m,p**) now lie below spiking threshold; this decreases the strength of interneuron activation required for subtraction of the inputs to appear division-like in the downstream neuron’s net input (**p–r**). This decreases the range over which activation/inactivation will produce consistent conclusions. **DOI:** http://dx.doi.org/10.7554/eLife.18383.025

This contrasts with the effect of interneuron activation. As we have previously demonstrated (Seybold et al., 2015) divisive suppression acts purely linearly on the weighted inputs (Figure 7i,j); from the perspective of a downstream neuron, the largest suppressive effect occurs near the center of its tuning curve. In contrast, subtractive suppression does not linearly suppress the firing of the input neurons because the weakest responses are suppressed below the nonlinear spiking threshold, creating a 'floor effect' at the edges of the tuning curve. In the downstream neuron, these nonlinear floor effects at the edges sum to produce the strongest suppression at the center of the tuning curve (Figure 7n), and thus, downstream, appear division-like (Figure 7k,n). Importantly, aspects of the network state such as its baseline firing rate determine the regime wherein such asymmetric effects between activation and inactivation will be observed (Figure 8). For example, in a network with high spontaneous activity (Figure 8j), interneurons must be activated more strongly in order to suppress their targets’ firing rates below the spiking threshold; thus the range of interneuron manipulation levels over which the neural circuit behaves linearly is relatively large (Figure 8l). On the other hand, in a network with low spontaneous activity, in which the edges of the tuning curves already lie below threshold (Figure 8p), even small additive increases in firing rates are impacted by threshold nonlinearities; thus the linear operating range of the network becomes relatively small (Figure 8r). These results highlight that the qualitative conclusions drawn from optogenetic manipulations are sensitive to both the details of the manipulation and the state of the circuit in which the manipulation is performed.

## Discussion

Resolving mechanisms of sensory processing at the level of cortical microcircuits will involve dissecting the operations performed by each interneuron subtype. The field cannot achieve this goal with correlative studies alone; causal manipulations are essential tools for conclusively linking interneuron subtypes to their respective cortical functions. Nonetheless, the assumptions and simplifications generally made when using these causal manipulations must be scrutinized. Given the surge in the use of molecular genetic tools to causally probe interneuron function, we tested whether one of the main assumptions underlying much of this work is true. Specifically, we tested the idea that differences in normal function between interneuron populations may be directly read out, and straightforwardly interpreted, by simply dialing interneuron activity up or down. In three different aspects of sensory processing within auditory cortex – the regulation of the gain, tuning, and information content of tone responses – we found that this intuitive assumption was not correct. Activation of Sst+ and Pvalb+ interneurons produced no significant differences in any of the three domains of sensory processing that we tested, even in cases where activation of interneurons caused equivalent and strong suppression, implying that Sst+ and Pvalb+ interneurons function equivalently in these aspects of sensory processing. In stark contrast, inactivation of Sst+ and Pvalb+ interneurons produced significant differences in all three domains: Pvalb+ cells appeared to increase information transfer through subtractive inhibition, whereas Sst+ cells appeared to provide divisive inhibition. That optogenetic activation and inactivation of interneurons did not produce symmetric results contradicts the intuitive notion that optogenetically increasing and decreasing interneuron activity necessarily strengthens and weakens, respectively, the functions these interneurons normally serve. How could optogenetic activation and inactivation of interneurons lead to these seemingly contradictory outcomes?

One reason could be that activation and inactivation of interneurons may differentially affect how the features of interneuron-specific inhibition are propagated through a network like the cortex. To help the reader reason through this phenomenon, we used a simple multilayered model to show that, when activating interneurons, network structure and threshold nonlinearities can convert either subtractive or divisive inhibition of a neuron’s local inputs into divisive inhibition of a network (Seybold et al., 2015); yet when inactivating interneurons, multiplication and addition of local inputs may bypass these threshold nonlinearities and preserve their linear effects at the network level (Figure 7). Whether such asymmetric effects will occur depends on both the level of interneuron manipulation and the baseline activity of the input neurons. At high baseline activity, when cells spend much of their time above threshold, moderate changes in firing do not interact with threshold nonlinearities and, thus, the range of manipulation levels that produces symmetric effects expands. On the other hand, at low baseline levels, cells spend much of their time below threshold, and even small changes in firing will interact with threshold nonlinearities; thus the range of symmetric effects decreases (Figure 8). Importantly, this work suggests that optogenetic manipulations (both activation and inactivation) can be used as tools to reveal the operating range over which the network behaves linearly versus nonlinearly, with regards to any neuronal computation. This work goes beyond the previous work of our and other groups (Douglas et al., 1995; Litwin-Kumar et al., 2016; Murphy and Miller, 2003; Seybold et al., 2015; Tsodyks et al., 1997) that identify discrepancies between the single-cell and network-level consequences of inhibition. Here our goal is different: to understand the interactions between increasing or decreasing inhibition and the innate properties of the network, as part of a larger program of clarifying the ways in which we can – and cannot – use optogenetic and other causal strategies to establish the functions of specific cell types.

Another confound that could contribute to the asymmetric results from our experiments is that bidirectional manipulations may target a single population of interneurons that is genetically homogeneous, yet contains functionally heterogeneous subpopulations that respond to sensory stimuli in distinct ways. This consideration goes beyond the widely appreciated one that existing genetic targets may encompass several genetically distinct cell types (Hu et al., 2013; Ma et al., 2006; Tasic et al., 2016). For example, from the viewpoint of a cortical pyramidal cell that receives inputs from several genetically similar interneurons, inactivation of these interneurons only suppresses neurons that are active during the stimulus. On the other hand, activation of these interneurons recruits most, if not all, of the interneurons – even those that are not normally activated by the stimulus. In this study, for example, a small population of the putative Sst+ and Pvalb+ interneurons fired weakly during the auditory stimulus (with a mean firing rate of less than 5 Hz), but dramatically increased their firing, often well above 30 Hz, with light stimulation. Moreover, recent work suggests that cortical Sst+ and Pvalb+ populations may contain subgroups that are differentially driven by patterns of cortical activity (Kwan and Dan, 2012), stimulus features (Runyan et al., 2010), and task-related behaviors (Kvitsiani et al., 2013; Lagler et al., 2016), suggesting that genetically-targeted activation of these cells may recruit more than one functional population. Thus, activation of these heterogeneous populations may strengthen different sets of functions than those weakened by their inactivation, leading to inconsistent conclusions regarding their normal function.

Other issues related to the normal pattern of interneuron activity may also contribute to these contradictory findings. Firstly, both the temporal organization of interneuron activity – for instance response delays (Gabernet et al., 2005; Tan et al., 2008; Wehr and Zador, 2003) or synchrony (Cardin et al., 2009; Hasenstaub et al., 2016; Hasenstaub et al., 2005; Hu and Agmon, 2015; Otte et al., 2010; Sohal et al., 2009; Stevens and Zador, 1998) – and the stimulus preferences of the interneurons themselves (Atencio and Schreiner, 2010; Kerlin et al., 2010; Li et al., 2015; Moore and Wehr, 2013) are important for determining output. For example, in auditory cortex, Pvalb+ cells have been reported to be activated rapidly and by a broad set of tones, while Sst+ interneurons respond relatively slowly to a narrower range of tones (Li et al., 2015). These features, however, will not be replicated in studies in which interneuron firing is indiscriminately and tonically increased or decreased. Additionally, activating or inactivating interneurons beyond a certain threshold may drive the cortical network beyond its natural operating regime (for instance, by breaking inhibitory-excitatory balance [Ozeki et al., 2009; van Vreeswijk and Sompolinsky, 1998], pushing the cortex towards epileptic states [Chagnac-Amitai and Connors, 1989], or close to silence [Hess and Murata, 1974]); in other words, a given change in interneuron activity may have a different effect in a normally active circuit compared to an over- or underactive circuit. Given these potential confounds, the results of this study further support the notion that the precise way in which we use causal tools, and the brain states in which the manipulations are performed, will govern the conclusions drawn about neural function.

These findings provide context for recent and future investigations of interneuron function. For example, recent studies in visual cortex in which interneurons were activated with ChR2 have produced diverse and even opposing findings regarding interneuron subtypes’ contributions to the gain and tuning of sensory responses (Atallah et al., 2012; Lee et al., 2012; Wilson et al., 2012). The findings from our model suggest that, in any of these studies, including our own, activation of Sst+ or Pvalb+ interneurons may have suppressed input cell firing below nonlinear spiking threshold, transforming, or obfuscating, the computational properties of interneuron-specific inhibition. Our inactivation experiments, on the other hand, mostly agree with (Lee et al., 2012), and support the idea that Pvalb+ cells subtractively suppress responses and increase information content by suppressing irrelevant spikes, whereas Sst+ cells divisively suppress responses to modulate dynamic range. However, we stress the caveat that both cell types may perform these operations in cooperation with other cell types (Karnani et al., 2016; Lee et al., 2013; Pfeffer et al., 2013; Pi et al., 2013; Xu et al., 2013) and through complex network dynamics that we have not identified here (Litwin-Kumar et al., 2016). Moreover, although it is often proposed that activation and inactivation of neuronal populations test their necessity and sufficiency, respectively, to support certain computations or drive specific behaviors, our results, along with previous work (rev. [Allen et al., 2015]), suggest that this interpretation may suffer from the same second- and higher-order network effects as described here.

That we should be cautious in interpreting the effects of causal manipulations is not a novel idea. Recently, it has been shown that transient inactivations may not replicate the results of permanent lesion studies (Otchy et al., 2015). This can occur because brief manipulations in one brain region may transiently perturb the computations of downstream circuits and, thus, may not reveal circuit function within a steady-state. Our finding that opposing optogenetic manipulations can produce apparently paradoxical conclusions regarding cell-type function adds to the growing sentiment that the ease with which manipulation experiments can be performed belies the difficulty of their interpretation. We propose that activation may be particularly likely to mask normal interneuron function because it targets interneurons that are not normally active during a stimulus response, and it is likely to obscure distinctions between linear transformations within a nonlinear network; conversely, inactivation targets only the normally active subset of interneurons and is more likely to preserve distinctions between linear transformation types within a nonlinear network.To dissect how interneurons act cooperatively or individually to control cortical function, even modern causal manipulations must be carefully designed and interpreted.

## Materials and methods

### Animals

All experiments were approved by the Institutional Animal Care and Use Committee at the University of California, San Francisco. To target our opsins to either Sst+ or Pvalb+ cells, we used Sst-Cre and Pvalb-Cre knock-in lines (JAX strains 013044/RRID:IMSR_JAX:013044 and 008069/RRID:IMSR_JAX:008069, with mixed C57BL/6;129S4 and C57BL/6;129P2 backgrounds, respectively). These strains drive expression of Cre in Sst+ and Pvalb+ interneurons of the cortex and hippocampus with minimal (<10%) leak (Taniguchi et al., 2011). To produce mice in which we could either inactivate or activate interneurons, we crossed these Cre lines to either the Ai35 or Ai32 lines (JAX strains 012735/RRID:IMSR_JAX:012735 and 012569/RRID:IMSR_JAX:012569 with C57BL/6 backgrounds), which, respectively, encode the light-gated hyperpolarizing proton pump Archaerhodopsin-3 (Arch) conjugated to GFP, or the light-gated depolarizing cation channel channelrhodopsin-2 (ChR2) conjugated to eYFP, after a floxed stop cassette under the CAG promoter. For all experiments, we used adult male or female mice that were 6 to 12 weeks old. All adult mice were housed in groups of 2–5 under a 12 hr/12 hr light/dark cycle.

### Histology

Adult mice were deeply anesthetized with ketamine/xylazine and perfused transcardially with 4% paraformaldehyde (PFA) in PBS (0.1 M, pH 7.4). After removal, brains were post-fixed overnight in the same PFA/PBS solution and then transferred to a solution of 30% sucrose 0.1 M PBS until they sank to the bottom of the tube. Coronal sections (40 μm thick) were cut using a freezing microtome and placed into a cryo-protective solution (30% ethylene glycol, 30% glycerol, in 0.1 M PBS). Slices were rinsed in PBS solution three times for 10 min, and then in 0.25% Triton X-100/0.1 M PBS three times for 10 min. After rinsing, slices were incubated in blocking solution (0.25% Triton X-100 and 10% normal donkey serum in 0.1 M PBS) for 2 hr, and then overnight at 4°C in the primary antibodies: chicken-anti-GFP (1:500, GFP-1020 Aves Lab RRID:AB_10000240), and either rabbit anti-parvalbumin (1:1000, RRID:AB_10013386 Swant, Switzerland) or goat anti-somatostatin (1:300, SC-7819 RRID:AB_2302603 Santa Cruz Biotechnology, Inc., Dallas, TX) diluted in 0.25% Triton X-100, 10% normal donkey serum, in 0.1 M PBS. Sections were then rinsed in blocking solution of 5% normal donkey serum, 0.25% Triton in 0.1 M PBS three times for 10 min and incubated in the same blocking buffer for 2 hr, with secondary antibodies: donkey-anti-chicken-Alexa 488 (1:200, 703-545-155 RRID:AB_2340375 Jackson ImmunoResearch, West Grove, PA), and either donkey anti-rabbit-Alexa 594 (1:200, 711-585-152 RRID:AB_2340621 Jackson ImmunoResearch) or donkey anti-goat-Alexa 594 (1:200, 705-586-147 RRID:AB_2340434 Jackson ImmunoResearch). The sections were then rinsed in 0.1 M PBS three times for 10 min, mounted on gelatin-subbed slides, and allowed to dry. Then they were dehydrated and defatted with 50% ethanol for 2 min, 70% ethanol for 2 min, 95% ethanol for 5 min, 100% ethanol for 10 min, 100% ethanol for 10 min, xylenes for 10 min, and xylenes for 10 min. The sections were immediately coverslipped using Krystalon mounting medium (EMD Millipore, Gibbstown, NJ) and dried overnight. Digitized images were obtained with a Nikon DS-Fi1 digital camera (Nikon Instruments, Melville, NY) on a Nikon ECLIPSE 90i microscope (Nikon Instruments) using a 10× or 20× objective.

### In vivo awake recordings

An initial surgery was performed 1–5 days prior to recording to affix a custom metal headplate over the temporal skull with dental cement. On the day of recording, a second surgery was performed to expose the auditory cortex. For both surgeries, mice were anesthetized with isoflurane and a subcutaneous injection of lidocaine under the incision site. As a post-operative analgesic, mice were given a subcutaneous injection of carprofen. An opening in the skull (∼2 mm) centered over auditory cortex was drilled and then filled with silicone elastomer to protect the brain. Animals were allowed to recover from the anesthesia for 1–3 hr, after which they were placed in a head holder on a free-spinning spherical treadmill (modified from [Niell and Stryker, 2008]), after which the silicone plug was removed. Recordings were made using a 16 site linear probe (50 μm spacing, Neuronexus), inserted perpendicular to the cortical surface. Stimuli consisted of randomly ordered 20 or 50 ms tones of various frequencies (4 kHz to 64 kHz, 0.2 octave spacing, 1 s interstimulus interval, with the intensity level chosen as 10–15 dB above threshold at BF) presented through a free-field high-frequency speaker (ES1, Tucker-Davis Technologies, Alachua, FL). In most experiments a second tone was played either 50 or 70 ms after the onset of the first tone for a different set of analyses. Thus only the first 50 ms of the first tone response was analyzed.

On randomly interleaved trials, the cortical surface just above the probe was illuminated with blue light (in ChR2 mice) or green light (in Arch mice) through a 400-μm-diameter fiber optic connected to a 470 nm LED (Mightex, Pleasanton, CA) or a 532 nm laser (IkeCool, Anaheim, CA), respectively. Recordings with blue light were performed with a light power near 15 μW (range 1 to 25 μW), which typically suppressed firing to about 50% of the light-off condition. Recordings with green light were performed with a light power near 10 mW (range 10 to 15 mW), which typically increased firing to about 150% of the light-off condition. The light began at least 50 ms (up to 250 ms) before the tone onset, linearly ramped up to full power for 50 ms, and remained on until 100 ms after the end of the stimulus. Each stimulus was presented 10–50 times with and without light. Responses were amplified and digitized continuously with a 16-channel recording system (Tucker-Davis Technologies) at 24,414 Hz. Events in the recordings that crossed a threshold of 4 SD were collected and sorted using custom software in MATLAB (KFMMAutosorter, written by Matthew Fellows) to identify single units.

### Data analysis

Data were analyzed in MATLAB (MathWorks, Natick, MA). Spike waveforms were characterized by trough-to-peak duration. The distribution of trough-to-peak durations was bimodal (Figure 3—figure supplement 2 and Figure 5—figure supplement 2), allowing us to group unit waveforms into narrow-spiking (≤450 μs) or broad-spiking (>450 μs) categories. Event rasters for each unit were constructed around each tone and used to produce frequency tuning curves. The response onset was calculated as the time a unit’s response reached ¼ of the maximum response, and the frequency tuning curve for each unit was defined to be the firing rate during the period between this calculated response onset and 50 ms after it, as a function of frequency. A unit was defined as tuned if the frequency tuning curve was significantly (α = 0.05) modulated by frequency, calculated using a 1-way ANOVA, and if at least three responses in both the light-off and light-on conditions were significantly (α = 0.05) above zero. The firing rate suppression or enhancement ratio for each unit was calculated as the percent change in spikes-per-trial during the response period, averaged over all stimuli. A unit was considered significantly suppressed or enhanced by the light if the number of tone-evoked spikes during the 50 ms response period across all stimulus conditions with light was significantly (α < 0.05) different from that without light. For Figure 3—figure supplement 3 and Figure 5—figure supplement 3, we calculated baseline-subtracted tone-evoked firing by subtracting the average spontaneous firing rate, measured from 0 to 50 ms before stimulus onset, from the tone-evoked rates. For Figure 3—figure supplement 5 and Figure 5—figure supplement 5, units from either ChR2/Sst and ChR2/Pvalb mice, or Arch/Sst and Arch/Pvalb mice were matched based on their firing rate ratios by selecting pairs of units between the two groups that were within a distance of a tenth of the range of all firing rate ratios. If a unit in one group did not have a matching unit in the other group within this distance, it was discarded. We analyzed linear transformations using standardized major axis regression, which is necessary to account for the measurement variance on both the x and y axis, which ordinary least-squares regression does not (Sokal and Rohlf, 2012). Only stimuli that elicited a firing rate significantly above zero in both light-off and light-on conditions were included in the analysis to avoid floor effects on spiking activity which could bias the regression and cause a subtractive effect to appear divisive. Units were deemed to show significant divisive or multiplicative transformations if the regression slope was significantly (α = 0.05) less than one or greater than one, respectively. Units were deemed to show significant subtractive or additive transformations if the regression y-intercept was significantly (α = 0.05) less than zero or greater than zero, respectively.

Per-trial mutual information (MI) for each unit was calculated using the formula:

$$I\left(X;Y\right)=\;\underset{y\in Y}{\sum }\underset{x\in X}{\sum }p\left(x,y\right)*log\left(\frac{p(x,y)}{p\left(x\right)*p(y)}\right)$$

where x represents possible values for the first variable (e.g., the frequency of the tonal stimulus), y represents possible values for the second variable (e.g., the number of output spikes), p(x) is the probability of observing x, p(y) is the probability of observing y, and p(x,y) is the joint probability of the two variables (i.e., the probability of observing xstimulus and y spikes). Because we have a limited number of trials from which to sample the stimulus and response probability distributions, MI might be upwardly biased. To account for this bias, we repeatedly (500 times) shuffled stimulus-response pairs, such that each response was associated with a randomly chosen stimulus, thus eliminating information due to a real relationship between the stimulus and response, but retaining information due to a bias. We then subtracted the average of the shuffled MI values from the originally measured MI to obtain the bias-corrected MI. We applied this method to both light-off and light-on conditions separately. To calculate information-per-spike for each unit, we divided the information-per-trial by the average firing rate (spikes-per-trial).

### Statistics

Unless otherwise stated, all statistical values were calculated in MATLAB. Unless otherwise noted, distributions were plotted with boxplots, where the box represents the first quartile, the second quartile (median) and the third quartile of the data, the whiskers represent 1.5*interquartile range (third-first quartiles), and the dots represents outliers lying beyond the whiskers. Statistical descriptions of distributions for the putative interneurons were reported as the median ± median absolute deviation.

Significance of regression parameters for each unit was determined based on whether they exceeded the 95% confidence bounds, as in (Sokal and Rohlf, 2012). To determine whether changes in MI were significant for each unit, we performed a bootstrap analysis: we repeatedly (500 times) randomly reassigned trials to the light-off and light-on conditions and recalculated the response metric for each reassignment. Effects were deemed significant if the observed effects were less than 2.5% or greater than 97.5% of the bootstrap-calculated distribution of effects. We used Wilcoxon sign-rank test to determine whether light significantly affected a population of units, Wilcoxon rank-sum test to determine whether continuous parameters were differentially distributed between groups, and a Fisher’s exact test (calculated in R) to determine if the distributions of all linear transformation types were significantly different between groups. All tests were two-sided.

### Model

We assumed a population of N frequency-tuned input neurons $I_{n}$, each with a Gaussian tuning curve, systematically varying in their center frequency:

$$I_{n}\left(f\right)=e^{-\left(\frac{{\left(f-n\right)}^{2}}{2*\sigma_{I}{}^{2}}\right)}$$

These input neurons are connected to the target neuron by a center-weighted (Gaussian) connectivity function W:

$$W\left(x\right)=e^{-\left(\frac{x^{2}}{2\sigma_{W}^{2}}\right)}$$

The target neuron’s total drive $I_{net}$, as a function of frequency, is then

$$I_{net}\left(f\right)=\underset{n}{\sum }I_{n}(f)W(n)$$

The target neuron was assumed to be threshold-linear (i.e., its firing rate is proportional to its input, except that subthreshold inputs produce a firing rate of zero). Its output O is calculated by thresholding its total input against a threshold T:

$$O_{T}\left(f\right)=\text{max}(0,I_{net}(f)-T)$$

When input neurons are partially suppressed or enhanced, each input neuron’s activity is calculated as:

$$I_{n}^{lion}\left(f\right)=\text{max}(0,m\;I_{n}^{lioff}(f)-b)$$

Here m and b represent the strengths of divisive/multiplicative and subtractive/additive inhibition, respectively. The target neuron’s net drive, output, and change in responsiveness are then calculated as:

$$I_{net}^{lion}\left(f\right)=\underset{n}{\sum }I_{n}^{lion}\left(f\right)W\left(n\right)$$

$$O_{T}^{lion}\left(f\right)=\text{max}(0,I_{net}^{lion}(f)-T)$$

$$\Delta O\left(f\right)=O_{T}^{lioff}\left(f\right)-O_{T}^{lion}\left(f\right)$$

For all conditions (division, multiplication, subtraction, and addition), 101 neurons provided input to the downstream neuron, whose tuning curves had center frequencies linearly spaced from −5 to 5, relative to that of the downstream neuron, and with standard deviation of 1. Unless otherwise stated, spiking threshold was set to 0. The connection weights of these inputs onto the downstream neuron decreased from a maximum of 0.2, at the best frequency of the downstream neuron, according to a Gaussian connectivity function with a standard deviation of 2. Divisive inhibition was then modeled by multiplying the input tuning curves by 0.5, while multiplication was modeled by multiplying the input tuning curves by 1.5. We modeled subtractive and additive changes in firing by subtracting and adding, respectively, 0.15 from the entire tuning curve of each input neuron. In Figure 8, the baseline firing of the input neurons was varied by changing the spiking threshold (i.e., by adding or subtracting a constant to each Gaussian input curve). For moderate baseline firing, threshold remained at 0, for low baseline firing threshold was set to 0.1 and for high baseline firing the threshold was set to −0.1.

### Data reporting

The sample size of units was chosen to be consistent with similar studies in the field. The experimenter was not blinded to the genotype of the animal during recordings but was blinded to the genotype of the animal during the spike sorting process.
