# Supplementary material for: Asymmetric effects of activating and inactivating cortical interneurons
Source: eLife. 2016 Oct 10;5:e18383. doi: 10.7554/eLife.18383 (PMC5123863; doi:10.7554/eLife.18383)
Supplement: Related texts

# Article commentary: Decision letter

**DOI:** 10.7554/eLife.18383.026

**Affiliations:** Harvard University, United States

In the interests of transparency, eLife includes the editorial decision letter and accompanying author responses. A lightly edited version of the letter sent to the authors after peer review is shown, indicating the most substantive concerns; minor comments are not usually included.

Thank you for submitting your article "Asymmetric Effects of Activating and Inactivating Cortical Interneurons" for consideration by *eLife*. Your article has been favorably evaluated by Timothy Behrens (Senior Editor) and three reviewers, one of whom, Naoshige Uchida (Reviewer #1), is a member of our Board of Reviewing Editors. The following individuals involved in review of your submission have agreed to reveal their identity: Maria Neimark Geffen (Reviewer #2); Andrea Benucci (Reviewer #3).

The reviewers have discussed the reviews with one another and the Reviewing Editor has drafted this decision to help you prepare a revised submission.

Summary:

The authors examined how activation and inactivation of specific types of interneurons in the auditory cortex affect gain, frequency tuning, or information content of tone-evoked responses in excitatory neurons. The authors found that activation of interneurons did not produce cell-type specific effects, but inactivation caused cell-type specific effect in all three measures. The authors also include a simple multilayered network model to show how subtraction and addition could produce these asymmetrical effects.

All the referees found that this is a timely and important study demonstrating some caveats in interpretations of experiments involving transient inactivation. The manuscript is well-written and the authors make careful discussions.

While all the referees were enthusiastic, there are some issues to which we would like to see your response. We therefore would like to invite you to revise the manuscript.

Essential points:

1) One main issue that was common in the reviewers' comments is considering the normal operating regime of neural circuit to design and interpret experiments. First, can the authors mention how PV and SST interneurons fire in a normal condition (reviewer #1, point #1)? If the authors have relevant data or if there are some literature on these, it would be great to describe them more clearly. Second, it seems that the authors have conducted a number of analyses to control for varying effects of optogenetic stimulation on different neurons during the tone responses. How do the effects of optogenetic stimulation on spontaneous activity compare to effects on tone-evoked responses? Would it be possible to quantify tone response magnitude rather than firing rate, which may be the more relevant quantity used by the brain to make behavioral decisions (reviewer #2, point #2)? Related to this issue, have the authors looked at the effect of varying intensity of laser stimulations? Third, it appears that one way to look at the experiments is that they are testing the dynamic range at which the neural circuits operate in a linear regime with regard to gain, frequency tuning, or information content of tone-evoked responses (reviewer #3, point #2). More detailed comments on each of these points can be found in the individual referees' comments appended below. Based on these, please make coherent discussions on the dynamics of neural circuits, model, and the experimental results.

2) Please quantify the specificity of Arch/ChR2 expression in SSTs (reviewer #2, point #1).

3) Please clarify how the authors selected neurons for analysis. Can the authors select putatively pyramidal or wide-spiking neurons for analysis (reviewer #1, point #1)?

*Reviewer #1:*

This study compared the effects of optogenetically inactivating versus activating specific types of inhibitory interneurons, pervalbumin (PV)- or somatostatin (SST)-expressing interneurons, on the tuning curve of neurons in the primary auditory cortex in mice. To quantify whether these manipulations caused divisive or subtractive changes in tuning curves, responses in light-on trials were regressed by responses in light-off trials. Divisive changes were detected as a significant change in slope whereas subtractive changes were detected as a significant change in intercept. Furthermore, the authors compared the ability of each neuron to transmit stimulus information using mutual information between the two conditions (activation versus inactivation). The authors' results showed that activating and inactivating specific interneurons often caused inconsistent results across these two conditions. The authors further explored potential mechanisms underlying these discrepancies using a simple network model, suggesting that some nonlinearity such as flooring of firing rates may explain these discrepancies. The authors discuss other potential caveats of their and other experiments in Discussion.

Although optogenetic as well as other manipulations such as pharmacological, pharmaco- and magneto-genetic manipulations plays critical roles in elucidating the role of specific neuronal populations, various caveats in interpreting these results have not been fully appreciated. This study adds a timely and important warning to the neuroscience community. The results are interesting and presented clearly. Although there are some points that need to be clarified, I believe this study warrants publication at *eLife*.

1) How do PV and SST neurons respond to different stimuli with or without optogenetic manipulations? What are the time courses and what are their tuning curves of these interneurons during auditory stimulation? These types of information are very important in designing optogenetic stimulation parameters (such as the timing and magnitude of manipulations, as the authors discuss). Also, one important assumption of these experiments appears to be that each neuronal population shows homogeneous (or similar) responses across stimuli and neurons (as neurons were homogeneously manipulated by optogenetics). Is this true to begin with?

2) It is important to know how the authors selected neurons for their analysis (beyond focusing on those that changed their firing). Ideally, the analyses should be separately performed for putative pyramidal and interneurons, and further, PV and SST neurons. Even though the classification might not be perfect, can the authors analyze subsets of their data that belong putatively to specific neuron types?

*Reviewer #2:*

In this important, timely, and elegant study, the authors demonstrate that activating and inactivating two different interneuron subtypes in the auditory cortex has asymmetrical effects on gain, frequency tuning, and information content of neural responses. Specifically, that optogenetic activation of interneurons did not produce cell-type specific effects on gain, frequency tuning, or information content of tone-evoked responses in excitatory neurons, but optogenetic inactivation of interneurons caused cell-type specific effect in all three measures. The effects on putative excitatory neuronal responses to tones were multiplicative with inactivation of SOMs and additive with inactivation of PVs, whereas activation of either PVs or SOMs led to a mix of subtractive and divisive effects. The latter result is consistent with a recent paper by the authors (Seybold et al., Neuron, 2015), whereas the former is novel to this manuscript. The authors also include a simple multilayered network model to show how subtraction and addition could produce these asymmetrical effects.

I have the following suggestions:

1) The evidence for Arch/ChR2 expression in SSTs seems to be missing. While Arch/ChR2 does not co-localize with PV, that does not necessarily mean that it is expressed in SSTs, and exclusively in SSTs. Quantifying this is essential to interpreting the results from the paper, which rely on the comparison between the effects of activating SSTs and PVs. Please provide a picture of the stain for somatostatin and demonstrate co-localization of Arch and ChR2 expression and som. Please provide quantitative data for co-localization (specificity and efficiency).

2) It seems that the authors have conducted a number of analyses to control for varying effects of optogenetic stimulation on different neurons during the tone responses. How do the effects of optogenetic stimulation on spontaneous activity compare to effects on tone-evoked responses? Would it be possible to quantify tone response magnitude rather than firing rate, which may be the more relevant quantity used by the brain to make behavioral decisions?

3) The model is intuitive and easy to understand. However, it seems to be an oversimplification to exclude excitatory/inhibitory dynamics, and it would be beneficial to make the model more biologically relevant by examining rate dynamics for excitatory and inhibitory populations. Optogenetic manipulation of PVs and SSTs is represented as a multiplicative or divisive shift in the input units. It would be interesting whether this effect can be modeled through rate equations in which the optogenetic inputs would be represented as enhanced drive to inhibitory neuronal populations. This would also be interesting to examine w/r varying light intensity or varying magnitude of the effect of laser on spontaneous activity.

*Reviewer #3:*

The paper "Asymmetric Effects of Activating and Inactivating Cortical Interneurons" by Elizabeth Phillips and Andrea Hasenstaub emphasizes how the results of optogenetic activation and inactivation of interneurons are difficult to relate to the "natural" computations implemented by the population they belong to. In my view this is a fair warning worth disseminating among the optogenetic community. The manuscript is easy to read, and the data nicely presented. However, I have three concerns:

1) The main motivating argument of the study, that optogenetic activation is commonly believed to strengthen computation and inactivation to weaken it, is questionable. I think there is a widespread understanding that inactivation at most can probe the 'necessity' of a population in a given information-processing context, while activation can probe the 'sufficiency'. In my view, a preferable motivation would be the one presented in the Discussion (last paragraph) about the use of optogenetics to characterize linear regimes of information processing within non-linear networks.

2) The demonstration of "separable effects" or "asymmetric results" following activation or inactivation of interneurons, if taken literally in its generality, is a fairly well-understood concept. Instead, I believe the authors wanted to focus on the role of interneurons within physiological dynamical ranges (e.g. away from epileptic states, when completely silencing PV+ interneurons, or from an unresponsive network, when strongly driving PV+ interneurons), and on how optogenetic perturbations can shed light onto the linearity (or lack of thereof) of gain changes, tuning, and information content of the network. I would make this point more explicit.

3) A significant amount of (similar) data and analyses shown here have already been presented in their Neuron 2015 Viewpoint paper. Figures 1–8 in that paper cover most of the results shown here for optogenetic activation of SST+ and PV+. Similarly, a key point presented here regarding the subtractive suppression and its flooring effect (Figure 7M) was already made in Figure 8K in the 2015 paper. The novelty of this work is about the inactivation experiments, and I think this should be more explicit.

[Editors' note: further revisions were requested prior to acceptance, as described below.]

Thank you for submitting your article "Asymmetric Effects of Activating and Inactivating Cortical Interneurons" for consideration by *eLife*. Your article has been favorably evaluated by Timothy Behrens (Senior Editor) and three reviewers, one of whom, Naoshige Uchida (Reviewer #1), is a member of our Board of Reviewing Editors. The following individuals involved in review of your submission have agreed to reveal their identity: Maria Neimark Geffen (Reviewer #2); Andrea Benucci (Reviewer #3).

The reviewers have discussed the reviews with one another and the Reviewing Editor has drafted this decision to help you prepare a revised submission.

Summary:

The authors have been responsive to the referees' comments, and overall the manuscript has been improved significantly.

There are some issues that need to be revised or clarified. Please respond the comments appended below. These issues are relatively minor.

*Reviewer #1:*

The authors have performed new analyses and modified the manuscript. These changes have improved the manuscript significantly, and addressed most of my concerns.

*Reviewer #2:*

The authors did a good job addressing the concerns of the reviewers and the manuscript is much improved.

*Reviewer #3:*

The authors have addressed my concerns and overall the manuscript has significantly improved. The concern on the linear/non-linear operating range has been nicely addressed with the new Figure 8, describing "consistent or paradoxical" effects depending on baseline activity. Although I could grasp the general idea behind this model simulation, it was not easy to find the exact details. Apologies if I missed it, but for example I could not find the difference between moderate-high-low activities (e.g. a fractional change?). A few more details to allow for reproducibility of this simulations would be helpful.

# Reply: Author response

**DOI:** 10.7554/eLife.18383.027

[…]

*Essential points:*

*1) One main issue that was common in the reviewers' comments is considering the normal operating regime of neural circuit to design and interpret experiments. First, can the authors mention how PV and SST interneurons fire in a normal condition (reviewer #1, point #1)? If the authors have relevant data or if there are some literature on these, it would be great to describe them more clearly.*

We identified 24 putative SST+ interneurons and 27 putative PV+ interneurons by their increased sound-evoked firing rates with light activation. We have added a summary of the following key response features for these units: 1) baseline firing rate, 2) evoked firing rate, 3) change in baseline firing with light activation, and 4) change in evoked firing with light activation in the last paragraph of the subsection “Optogenetic activation does not produce cell type specific effects on gain and tuning”. A new FigureFigure 5—figure supplement 6 shows examples of these effects. We have added references to literature on their firing properties in the fourth paragraph of the Discussion.

We have also provided commentary on how the optogenetic activation we performed relates to the cells’ normal function (Results: subsection “Optogenetic activation does not produce cell type specific effects on gain and tuning”, last paragraph and Discussion): specifically that 1) baseline firing rates increased proportionally more than evoked firing rates, suggesting that optogenetic activation increases the relative number of stimulus-independent spikes, potentially masking normal stimulus-driven function, 2) even interneurons that were not driven by the sound were strongly driven by optogenetic activation, meaning that different functional populations of interneurons were active in our manipulation compared to natural conditions, and 3) that specific response features of SST+ and PV+ interneurons identified in previous work, such as their response timing and tuning, may not be replicated in studies in which their activity is broadly and tonically manipulated. This lets us highlight what we agree is an essential point: that the conclusions one draws regarding a cell’s role in computation may be affected by how well the manipulation replicates the true response features of the cells themselves.

*Second, it seems that the authors have conducted a number of analyses to control for varying effects of optogenetic stimulation on different neurons during the tone responses. How do the effects of optogenetic stimulation on spontaneous activity compare to effects on tone-evoked responses?*

We have added a new Figure 1—figure supplement 2 and Figure 5—figure supplement 2, in which we quantify the effects of interneuron inactivation/activation on spontaneous firing rates, tone-evoked responses, baseline-subtracted firing rates, and signal-to-noise ratio (SNR: evoked rate divided by spontaneous rate). These figures show that optogenetic inactivation of interneurons on average increases their targets’ baseline firing more than evoked firing, thus reducing SNR, while optogenetic activation of interneurons on average decreases their targets’ baseline firing more than evoked firing, increasing SNR.

*Would it be possible to quantify tone response magnitude rather than firing rate, which may be the more relevant quantity used by the brain to make behavioral decisions (reviewer #2, point #2)?*

We have added a new Figure 3—figure supplement 3 and Figure 5—figure supplement 3, and accompanying text in the Results (subsection “Optogenetic inactivation produces cell type specific effects on gain and tuning”, first paragraph and subsection “Optogenetic activation does not produce cell type specific effects on gain and tuning”, third paragraph), in which we repeat our main regression analysis using baseline-subtracted firing rates rather than raw firing rates. Interestingly, the results are consistent with those obtained when the analysis is performed on the raw sound-evoked responses: inactivating PV+ cells still produces significantly larger additive effects, inactivating SST+ cells still produces significantly larger multiplicative effects, while activating PV+ and SST+ cells still produce similar distributions of effects. We appreciate this request because it revealed that the additive effects produced by inactivation of PV+ cells could not entirely be explained by increases in baseline activity, but must have included a substantial contribution from sound-evoked responses that were not frequency selective.

*Related to this issue, have the authors looked at the effect of varying intensity of laser stimulations?*

No, unfortunately we did not systematically vary the intensity of laser stimulation during these experiments. We did analyze, on a cell-by-cell basis, whether the presence of a significant additive/subtractive or multiplicative/divisive effect correlated with the strength of suppression in individual cells. This analysis is shown in Figure 3—figure supplement 4 and Figure 5—figure supplement 4. We found that the same response features (stronger addition with PV+ inactivation, stronger multiplication with SST+ inactivation, and similar effects with PV+ activation as with SST+ activation) were present both for strongly affected cells and for weakly affected cells.

We also included a new modeling figure, Figure 8, in which we systematically varied the strength of inhibition in the model (effectively, varying laser intensity) to let us discuss the relationship between the quantitative strength of the manipulation and the qualitative character of the resulting computation. We discuss this model further in our next response below.

*Third, it appears that one way to look at the experiments is that they are testing the dynamic range at which the neural circuits operate in a linear regime with regard to gain, frequency tuning, or information content of tone-evoked responses (reviewer #3, point #2).*

We think this is an excellent point and thank the reviewers for urging us to emphasize it. We have included one new model-based figure (Figure 8), to make this point explicit. In Figure 8, we choose one configuration of the model, and show how varying light levels reveals the range over which the network operates in a linear regime. We then vary one model parameter (in this case, we examine several different baseline activity levels), and show how this interacts with light level, to show that the extent of the linear regime varies depending on baseline activity. This highlights the point that the qualitative conclusions drawn from optogenetic manipulations are sensitive to both the details of the manipulation and the network state in which the manipulation is performed. We have changed text in the Abstract, Introduction, Results, and Discussion to give this point prominence.

*More detailed comments on each of these points can be found in the individual referees' comments appended below. Based on these, please make coherent discussions on the dynamics of neural circuits, model, and the experimental results.*

*2) Please quantify the specificity of Arch/ChR2 expression in SSTs (reviewer #2, point #1).*

We have supplied images of a SST stain in ChR2/SST and Arch/SST tissue in Figure 1—figure supplement 1 and Figure 4—figure supplement 1. The percentage of reporter-labeled neurons that showed SST immunofluorescence (i.e., specificity) was 89 ± 4% and the percentage of SST labeled neurons that were reporter-labeled (i.e., efficiency) was 91 ± 2%. This is consistent with the characterization of SST expression previously performed in Taniguchi et al. (2011), who found specificity of 92% ± 2% and efficiency of 93% ± 3%.

*3) Please clarify how the authors selected neurons for analysis. Can the authors select putatively pyramidal or wide-spiking neurons for analysis (reviewer #1, point #1)?*

Thank you for the opportunity to clarify this. We generally did not eliminate units from the analysis based on their action potential shape: instead, we analyzed all tone-responsive, frequency-tuned units whose firing was increased by light (for the interneuron inactivation experiments) or decreased by light (for the interneuron activation experiments). We have clarified this in the Results, subsection “Optogenetic inactivation of SST+ or PV+ interneurons”, last paragraph and subsection “Optogenetic inactivation produces cell type specific effects on gain and tuning”, first paragraph. We have also added two new figures, Figure 3—figure supplement 2 and Figure 5—figure supplement 2, in which we show the strength of multiplicative/divisive or additive/subtractive modulation as a function of spike width. We observe a bimodal distribution of spike durations, based on which we separate units into narrow-spiking (trough-to-peak duration of <0.45 ms) and broad-spiking (trough-to-peak duration of >0.45 ms) populations. Interestingly, the differences in linear effects with inactivation of SST+ cells versus PV+ cells were present in broad-spiking units, but not in narrow-spiking units (referenced in subsection “Optogenetic inactivation produces cell type specific effects on gain and tuning”, first paragraph). With activation of interneurons, neither broad-spiking nor narrow-spiking units showed differences in linear effects between activating SST+ or PV+ cells (referenced in subsection “Optogenetic activation does not produce cell type specific effects on gain and tuning”, third paragraph).

*Reviewer #1:*

[…]

*1) How do PV and SST neurons respond to different stimuli with or without optogenetic manipulations? What are the time courses and what are their tuning curves of these interneurons during auditory stimulation? These types of information are very important in designing optogenetic stimulation parameters (such as the timing and magnitude of manipulations, as the authors discuss). Also, one important assumption of these experiments appears to be that each neuronal population shows homogeneous (or similar) responses across stimuli and neurons (as neurons were homogeneously manipulated by optogenetics). Is this true to begin with?*

We have included a description of the response features of the putative interneurons we were able to record from, and how they were affected by light manipulation, in the paper (discussed under Essential points 1 above).

Regarding whether the neuronal populations show homogeneous responses, we thank you for giving us an additional chance to more directly address this point. We have added text in the Discussion (third paragraph) to comment on the heterogeneity of these genetically-defined interneuron types. In our experiments, we noticed that several putative interneurons did not respond strongly to auditory stimuli, while most did, arguing for functional heterogeneity within each genetically-identified population. Moreover, other recent in vivo work, suggests that several functional subpopulations, which are differentially driven by stimulus features or task-related behaviors, exist within each genetically-identified interneuron population. We agree that this functional heterogeneity complicates the interpretation of data from causal manipulations, especially when using activation in which the firing rate of nearly every genetically-targeted cell, regardless of functional subgroup, is affected.

*2) It is important to know how the authors selected neurons for their analysis (beyond focusing on those that changed their firing).*

We have clarified this in the first paragraph of the subsection “Optogenetic inactivation produces cell type specific effects on gain and tuning” and in the second paragraph of the subsection “Optogenetic activation does not produce cell type specific effects on gain and tuning”.

*Ideally, the analyses should be separately performed for putative pyramidal and interneurons, and further, PV and SST neurons. Even though the classification might not be perfect, can the authors analyze subsets of their data that belong putatively to specific neuron types?*

Yes. We performed the linear analysis separately for units with narrow- or broad-spiking waveform shape (discussed in detail under Essential points: 3 above). In the case of interneuron inactivation, we recorded relatively few neurons that we could unambiguously identify as opsin-expressing, but in the case of interneuron activation in which we could identify several light-activated cells, we have provided information regarding the effects of activation on their firing properties (discussed in more detail under Essential points: 1 above).

Reviewer #2:

*[…]*

*I have the following suggestions:*

*1) The evidence for Arch/ChR2 expression in SSTs seems to be missing. While Arch/ChR2 does not co-localize with PV, that does not necessarily mean that it is expressed in SSTs, and exclusively in SSTs. Quantifying this is essential to interpreting the results from the paper, which rely on the comparison between the effects of activating SSTs and PVs. Please provide a picture of the stain for somatostatin and demonstrate co-localization of Arch and ChR2 expression and som. Please provide quantitative data for co-localization (specificity and efficiency).*

We have provided images for the SST stains in Figure 1—figure supplement 1 and Figure 4—figure supplement 1, and have provided numbers for specificity and efficiency under Essential points: 2 above.

*2) It seems that the authors have conducted a number of analyses to control for varying effects of optogenetic stimulation on different neurons during the tone responses. How do the effects of optogenetic stimulation on spontaneous activity compare to effects on tone-evoked responses? Would it be possible to quantify tone response magnitude rather than firing rate, which may be the more relevant quantity used by the brain to make behavioral decisions?*

Yes, we have provided a new Figure 3—figure supplement 2 and Figure 5—figure supplement 2 addressing this question, and have discussed this in more detail under Essential points: 1 above.

*3) The model is intuitive and easy to understand. However, it seems to be an oversimplification to exclude excitatory/inhibitory dynamics, and it would be beneficial to make the model more biologically relevant by examining rate dynamics for excitatory and inhibitory populations. Optogenetic manipulation of PVs and SSTs is represented as a multiplicative or divisive shift in the input units. It would be interesting whether this effect can be modeled through rate equations in which the optogenetic inputs would be represented as enhanced drive to inhibitory neuronal populations. This would also be interesting to examine w/r varying light intensity or varying magnitude of the effect of laser on spontaneous activity.*

We agree that our model oversimplifies many aspects of actual cortical computation, including dynamics. The model is not intended to be complete; it is meant only as an easy-to-follow framework for reasoning about some of the contradictory ways in which optogenetic manipulations can interact with network properties to confound our interpretation of interneurons’ computational roles. Similar contradictions could likely be found in dynamical models.

*Reviewer #3:*

[…]

*1) The main motivating argument of the study, that optogenetic activation is commonly believed to strengthen computation and inactivation to weaken it, is questionable. I think there is a widespread understanding that inactivation at most can probe the 'necessity' of a population in a given information-processing context, while activation can probe the 'sufficiency'. In my view, a preferable motivation would be the one presented in the Discussion (last paragraph) about the use of optogenetics to characterize linear regimes of information processing within non-linear networks.*

Thank you for addressing the point of necessity and sufficiency; we have added a point of discussion on this in the fifth paragraph of the Discussion. We have also addressed the ability of optogenetics to characterize linear regimes in Figure 8 and in the second paragraph of the Discussion (discussed in more detail under Essential points: 1 above).

*2) The demonstration of "separable effects" or "asymmetric results" following activation or inactivation of interneurons, if taken literally in its generality, is a fairly well-understood concept. Instead, I believe the authors wanted to focus on the role of interneurons within physiological dynamical ranges (e.g. away from epileptic states, when completely silencing PV+ interneurons, or from an unresponsive network, when strongly driving PV+ interneurons), and on how optogenetic perturbations can shed light onto the linearity (or lack of thereof) of gain changes, tuning, and information content of the network. I would make this point more explicit.*

We have added text in the Abstract; Introduction, last paragraph; Results, last paragraph and Discussion, second and fourth paragraphs to make these points more explicit and have discussed this in more detail under Essential points: 1 above.

*3) A significant amount of (similar) data and analyses shown here have already been presented in their Neuron 2015 Viewpoint paper. Figures 1–8 in that paper cover most of the results shown here for optogenetic activation of SST+ and PV+. Similarly, a key point presented here regarding the subtractive suppression and its flooring effect (Figure 7M) was already made in Figure 8K in the 2015 paper. The novelty of this work is about the inactivation experiments, and I think this should be more explicit.*

Thank you for giving us the chance to clarify this. We have added text in the third paragraph of the subsection “Optogenetic activation does not produce cell type specific effects on gain and tuning”, and in the second paragraph of the subsection “Inactivation and activation produce asymmetric effects in a convergent network” to make this more explicit.

[Editors' note: further revisions were requested prior to acceptance, as described below.]

[…]

*Reviewer #3:*

*The authors have addressed my concerns and overall the manuscript has significantly improved. The concern on the linear/non-linear operating range has been nicely addressed with the new Figure 8, describing "consistent or paradoxical" effects depending on baseline activity. Although I could grasp the general idea behind this model simulation, it was not easy to find the exact details. Apologies if I missed it, but for example I could not find the difference between moderate-high-low activities (e.g. a fractional change?). A few more details to allow for reproducibility of this simulations would be helpful.*

Thank you for these positive comments. We have added further details on the exact parameters used as well as the baseline activity parameters in Figure 8 in the subsection “Data reporting” of the Methods and in the legends of Figures 7 and 8.
